# Limb-Bud and Heart (LBH) Upregulation in Cardiomyocytes under Hypoxia Promotes the Activation of Cardiac Fibroblasts via Exosome Secretion

**DOI:** 10.1155/2022/8939449

**Published:** 2022-09-06

**Authors:** Yuling Xu, Anbiao Wu, Jingyang Chen, Xudong Song, Minsheng Chen, Qicai Liu

**Affiliations:** ^1^Nanfang Hospital, The First School of Clinical Medicine, Southern Medical University, Guangzhou 510515, China; ^2^Guangdong Provincial Biomedical Engineering Technology Research Center for Cardiovascular Disease, Guangzhou 510280, China; ^3^Sino-Japanese Cooperation Platform for Translational Research in Heart Failure, Guangzhou 510280, China; ^4^Laboratory of Heart Center, Department of Cardiology, Zhujiang Hospital, Southern Medical University, Guangzhou 510280, China

## Abstract

The activation of cardiac fibroblasts (CFs) after myocardial infarction (MI) is essential for post-MI infarct healing, during which the regulation of transforming growth factor beta1 (TGF-*β*1) signaling is predominant. We have demonstrated that TGF-*β*1-mediated upregulation of LBH contributes to post-MI CF activation via modulating *α*B-crystallin (CRYAB), after being upregulated by TGF-*β*1. In this study, the effect of LBH-CRYAB signaling on the cardiac microenvironment via exosome communication and the corresponding mechanisms were investigated. The upregulation of LBH and CRYAB was verified in both cardiomyocytes (CMs) and CFs in hypoxic, post-MI peri-infarct tissues. CM-derived exosomes were isolated and identified, and LBH distribution was elevated in exosomes derived from LBH-upregulated CMs under hypoxia. Treatment with LBH+ exosomes promoted cellular proliferation, differentiation, and epithelial-mesenchymal transition-like processes in CFs. Additionally, in primary LBH^KO^ CFs, western blotting showed that LBH knockout partially inhibited TGF-*β*1-induced CF activation, while LBH-CRYAB signaling affected TGF-*β*1 expression and secretion through a positive feedback loop. The administration of a Smad3 phosphorylation inhibitor to LBH^KO^ CFs under TGF-*β*1 stimulation indicated that Smad3 phosphorylation partially accounted for TGF-*β*1-induced LBH upregulation. In conclusion, LBH upregulation in CMs in post-MI peri-infarct areas correlated with a hypoxic cardiac microenvironment and led to elevated exosomal LBH levels, promoting the activation of recipient CFs, which brings new insights into the studies and therapeutic strategies of post-MI cardiac repair.

## 1. Introduction

Myocardial infarction (MI) is considered as the major cause of heart failure (HF) [1], which results in high morbidity and mortality rates and raises increasing medical concerns [2]. Resident cardiac fibroblasts (CFs) are scattered in the healthy cardiac interstitium [3], while CF activation post-MI, which is characterized by fibroblast-myofibroblast transdifferentiation (FMT), accelerated proliferation, and extracellular matrix (ECM) accumulation, supports the myocardial structure [4], thus contributing to the post-MI healing of the infarcted myocardium. Additionally, activated CFs are considered to be more resistant to post-MI ischemic injury than cardiomyocytes (CMs), preventing further damage and rupture of the ventricular wall [5]. Previous studies have divided post-MI cardiac repair into three partially overlapping phases: the inflammatory phase, the proliferative phase, and the maturation phase [6]; excessive deposition of ECM mediated by activated CFs during the maturation phase, if left unchecked, promotes the pathogenesis of reactive fibrosis and cardiac remodeling in the remote myocardium, leading to ventricular dilation, HF, and even death [7, 8]. Therefore, in our opinion, attempts to potentiate post-MI cardiac repair should be focused on regulating the corresponding mechanisms during the inflammatory phase while preventing enhanced excessive fibrosis during the maturation phase.

The initial inflammatory phase post-MI is triggered by massive necrotic cell death and can be characterized by enhanced cardiac expression of proinflammatory cytokines that mediate the clearance of dead cells and ECM fragments to subsequently accommodate activated CFs [9]. For CF activation during the inflammatory phase, transforming growth factor beta 1 (TGF-*β*1) is considered as the major mediator [10, 11], promoting CF proliferation, FMT and collagen deposition. Limb-bud and heart (LBH) is a highly conserved transcriptional cofactor that has important roles in embryonic development and cardiogenesis [12]. In our previous study, aberrant LBH expression initiated by TGF-*β*1 stimulation was detected in CFs accumulating in the infarcted myocardium, which promoted CF activation via LBH-CRYAB signaling during post-MI infarct healing [13]. Additionally, the upregulation of hypoxia inducible factor-1*α* (HIF-1*α*) was triggered by hypoxic conditions during the inflammatory phase, which was related to LBH-induced CF proliferative acceleration [13], whereas CF activation is due to the comprehensive effects of different causes in the post-MI cardiac microenvironment [14], and potential LBH-associated mechanisms regarding the communication between CFs and other cardiac cell types, such as CMs, endothelial cells, inflammatory cells, and immune cells [15], remain largely unclear. In our preliminary experiment conducted on a mouse MI model, the upregulation of LBH and CRYAB observed in activated CFs during the inflammatory phase synchronized with the expressional changes in CMs in the peri-infarct myocardium, together with overexpressed HIF-1*α*. Here, we wondered whether the increased LBH level in CFs originated from LBH-upregulated CMs through intercellular communication.

The potential intercellular communication between CMs and CFs might be effectuated by the secretion of exosomes, which are nanoscale multivesicular bodies (MVBs) secreted by almost all cell types and serve as intercellular messengers mainly upon internalization by membrane fusion [16]. In the heart, exosomes can deliver functional elements (including DNA, RNA, proteins, and other metabolites) to neighboring or distant cells to reprogram the cardiac microenvironment, which is involved in the pathogenesis of various heart diseases [17]. Specifically, during the inflammatory phase of post-MI infarct healing, CM-derived exosomes have been reported to mediate the phagocytosis of dead cells in the infarcted myocardium [18] and to regulate other inflammatory responses implemented by infiltrating monocytes [19]. Additionally, LBH proteins can be transported by nasopharyngeal carcinoma– (NPC–) derived exosomes in both paracrine and autocrine manners, modulating the progression of NPC [20]. The relationship between the LBH gene and CM-derived exosomes, however, has not been studied and requires further examination.

Based on the information presented above and our results in previous studies, we hypothesized that in the peri-infarct myocardium, upregulated LBH in CMs during the inflammatory phase might be involved in LBH-CRYAB-mediated CF proliferation, differentiation into myofibroblasts (MCFs), and epithelial-mesenchymal transition– (EMT–) like processes through intercellular communication in the post-MI cardiac microenvironment, and our investigation in this study focused on CM exosome secretion.

## 2. Materials and Methods

### 2.1. Establishment of LBH-Knockout (KO) Mice and Animal Procedures

All animal procedures were designed and performed according to the regulations of the Institutional Animal Care and Use Ethics Committee of Zhujiang Hospital. The establishment of conventional LBH- KO C57BL/6 mice was performed by Cyagen, Inc., via the CRISPR/Cas9 technique (contract ID: KOAI200813MG1) to generate F_0_ founder animals; then, the wild-type (WT)/KO offspring were bred, identified, and maintained at the Animal Experiment Center of Zhujiang Hospital (Figure S1). The MI model was constructed according to our previous research [13]. All mice were sacrificed to obtain the hearts on days 2 and 3 after surgery. Each experimental group had at least four surviving mice for sampling at every time point. Paraffin-embedded tissue sections of the left ventricles were used for hematoxylin and eosin (H&E), Masson, and immunofluorescence staining (Figure S2), and the remaining peri-infarct tissues were used for western blot (WB) assays.

### 2.2. Isolation and Culture of Primary CMs and CFs

The isolation of primary CMs and CFs of neonatal Sprague-Dawley (SD) rats or neonatal LBH^KO^ C57BL/6 mice was performed according to a modified protocol [21]. Briefly, the left ventricles of neonatal mice (1-2 days) were rinsed in 0.25% EDTA-trypsin at 4 °C for 12 hours. After inactivation of trypsinization, the tissues were digested by 0.08% collagenase II at 37 °C under magnetic stirring; then, the cell suspension was placed into Petri dishes. After 2 hours of incubation, the medium was aspirated, and the attached cells were washed twice and cultured in complete DMEM (10% FBS, 1% penicillin-streptomycin), which was the general culture condition for the following treatment. The aspirated cell suspension was placed into new dishes for another 2 hours of incubation, then the medium containing unattached cells was centrifuged, and the pellets were resuspended and cultured in complete DMEM containing 1 mM 5-bromo-2′-deoxyuridine (BrdU) for 3 days until the attached CMs were pulsatile and were considered ready for the following treatment. Isolation of rat CFs followed the same protocol, and only the amounts of trypsin and collagenase II were modified accordingly. Immunofluorescence detection of myocardium markers, including cardiac myosin, cardiac troponin T (CTnT), and *α*-actinin-1 (ACTN1), was performed for each batch to identify the purities of the isolated CMs, while anti-vimentin, anti-collagen I, anti-*α*-smooth muscle actin (*α*-SMA), anti-PDGFR1, and anti-S100A4 were used to identify the purities of isolated CFs (Figure S3). For mouse/rat CFs, only cells from passage 1 were used for all the following assays.

### 2.3. Lentivirus Infection, siRNA Transfection, Cell Line Establishment, and Hypoxia Treatment

The lentiviruses Lv5-NC and Lv5-LBH (integrated with an eGFP reporter, GenePharma, Inc.) were used to infect mouse CFs separately at an optimized multiplicity of infection (MOI) of 50 in the presence of 5 *μ*g/ml polybrene. After 72 hours, the infection efficiencies were ensured by fluorescence microscopy (Figure S4). Knockdown of the CRYAB gene was achieved by siRNA transfection with liposomes (Lipofectamine 3000, Invitrogen) and was subsequently performed after transient infections with LBH lentivirus in CFs. The targeting sequence of the siRNA was selected based on Zhou's research [22]: sense (5′-CCAGGGAGUUCCACA GGAA-3′) dTdT and antisense (5′-UUCCUGUGGAACUCCCUGG-3′) dTdT. Additionally, the myocardial cell line H9c2 (purchased from ATCC) was infected with Lv5-NC or Lv5-LBH lentivirus and then subjected to 2 weeks of puromycin (2 *μ*g/ml) screening to obtain the stable H9c2 cell lines ectopically expressing the LBH gene (Figure S4). Primary mouse CMs and H9c2 cells underwent hypoxia treatment to mimic the post-MI cardiac microenvironment for *in vitro* detection. For hypoxic conditions, all myocardial cells were cultured in glucose-free, serum-free DMEM (Gibco™, #11966025) under 1% O2 (Forma™ Series 2 Incubator, Thermo Scientific) for 3 to 6 hours before the glucose-free culture medium was harvested for further assays. The time lengths were verified by morphological changes and western blotting, which were sufficient to induce cellular injury (Figure S5).

### 2.4. Exosome Isolation and Electron Microscopy

Exosomes were isolated from myocardial cell culture medium by a modified ultracentrifugation method (Figure S6). Briefly, when the myocardial cells reached 80% confluence, the culture medium was collected and centrifuged at 500 × *g* for 10 min, 2000 × *g* for 10 min, and 10,000 × *g* for 30 min at 4 °C (Thermo ST 16R Centrifuge). Then, the supernatants were centrifuged at 100,000 × *g* for 70 min at 4 °C twice (Beckman SW 32Ti Ultracentrifuge), and the pellets were resuspended in 100 *μ*l of PBS. Isolated exosomes were stored at − 80 °C and used within a week after isolation. Electron microscopic characterization was performed according to our previous research [13]. Exosomal markers were selected based on MISEV2018 guidelines [23, 24].

### 2.5. Nanoparticle Tracking Analysis (NTA) and Nanoflow Cytometry Measurement (NFCM)

Real-time characterization of the myocardial cell-derived exosomes was accomplished by a Zetaview system (Particle Metrix, Germany) and nanoflow cytometry (NanoFCM, China) [25]. For the size distribution measurement, samples equivalent to 20 *μ*g total protein were diluted 1,000 times for injection and analyzed by ZetaView version 8.04.02 software, which represented the results as the size distribution of the analyzed samples. For the quantification of target protein in exosomes, isolated samples were stained with fluorescently labeled antibodies RT for 30 min after being treated with permeabilization buffer (BD 554714, USA); then, the stained samples were recentrifuged twice at 100,000 × *g* for 30 min to remove any excess unbound antibody and applied to the nanoflow cytometry blank-calibrated by deionized water. The unstained testing samples were used as a negative control.

### 2.6. Exosome Labeling, Tracking and Internalization Assay

The culture medium of H9c2 cells transfected with the LBH-eGFP plasmid or pEGFP-C1 plasmid was collected for exosome isolation. The isolated exosome samples were subjected to nanoflow cytometry to detect potential LBH-eGFP fusion protein or supplemented into the culture medium for CFs to observe cellular uptake of the labeled exosomes. For simulation of the entire crosstalk process without exosome isolation, the coculture of LBH-eGFP-transfected H9c2 cells and rat CFs was implemented by Transwell inserts (Corning #3450, 0.4 *μ*m pore size) and glass bottom 6-well plates (Nest #801004). Also, the exosomes isolated from mouse CMs were labeled with PKH26 membrane dye (Sigma-Aldrich MINI26, USA) before being applied to the culture medium for CFs. After 6 hours of coculture with exosomes (20 *μ*g total protein for each 35 mm petri dish according to BCA assay) or 24 hours of coculture with H9c2 cells, the treated CFs were fixed with 4% paraformaldehyde (PFA), stained with phalloidin-rhodamine (Santa-Cruz PHDR1, USA)/phalloidin-FTIC (Sigma-Aldrich P5282, USA) and 4′,6-diamidino-2-phenylindole (DAPI, FluoroPure™ grade, Invitrogen), respectively, and imaged by a Leica SP8 confocal microscope to confirm the internalization of labeled exosomes and the intracellular distribution of LBH-eGFP. The 3D images were remodeled from z-stack series with the 3D viewer module of Las X software.

### 2.7. Plasmid Transfection, TGF-*β*1 Stimulation, and Inhibitor Treatments

The LBH-eGFP plasmid and the pEGFP-C1 plasmid as a negative control were transfected into the H9c2 cell line by liposomes (Lipofectamine 3000, Invitrogen) to track extracellular secretion of the LBH protein in isolated exosome samples, all according to the manufacturer's instructions. The transfected H9c2 cells were subjected to 2 weeks of G418 (0.4 *μ*g/ml) screening before subsequent experiments (Figure S4). For TGF-*β*1 stimulation, mouse CFs were first serum starved overnight (O/N) and then treated with TGF-*β*1 (10 ng/ml, R&D) for 36 hours, according to our previous research [13]. For treatment with the Smad3 phosphorylation inhibitor, 10 *μ*M inhibitor SIS3 (Selleck, S7959) was added into the culture medium of mouse CFs during TGF-*β*1 stimulation, which had been identified as a sufficient amount to achieve and sustain inhibitory function (Figure S7). The treated cells were harvested 36 hours later for further assays. For treatment with the exosome production inhibitor, 10 *μ*M inhibitor GW4869 (MCE, HY-19363) was added into the culture medium of H9c2-LBH-eGFP cells cocultured with rat CFs, which was verified to be the concentration sufficient enough to inhibit exosome production and secretion from H9c2 cells during the entire coculture process (Figure S8).

### 2.8. Cellular Proliferation Assays

Proliferation experiments were performed by CCK-8 assay, 5-ethynyl-2′-deoxyuridine (EdU) staining and immunofluorescence staining with anti-Ki67. The CCK-8 assay was performed using a CCK-8 assay kit (Dojindo, Inc.) with a Varioskan LUX plate reader (Thermo Scientific) based on a previously reported protocol [26]. EdU staining was performed with a BeyoClick™ EdU-555 assay kit (Beyotime Biotech) according to the manufacturer's instructions. The stained samples were imaged by a Leica DMi8 fluorescence microscope. The EdU positive rates and Ki67 positive rates were calculated by ImageJ software, and for every sample, the EdU/Ki67 positive rates of 6 random fields were used for statistics.

### 2.9. Cellular Migration Assay

The migration of CFs was measured by Transwell assays based on the procedures described in our previous research [13].

### 2.10. Western Blot (WB) Assay

Western blotting was performed as previously described [27]. Specifically, for exosome samples, the loading amount was adjusted to 60 *μ*g after the samples were concentrated with a freeze dryer (Chirst ALPHA 1-4 LD plus). The antibodies used are listed in Table S1. Membrane exposure was performed by ECL, and a GE ImageQuant LAS 500 exposure instrument, while quantification was performed by ImageJ software.

### 2.11. Immunofluorescence Staining

Immunofluorescence staining was performed as previously described [13]. All stained samples were mounted and then imaged by a Leica SP8 confocal fluorescence microscope. Whole-slide images of mounted tissue sections were photographed by a GE Amersham Typhoon imager. The in cell western (ICW) assay was performed in multichamber slides (NUNC #154526) with a modified protocol based on the procedures of Egorina et al. [28] and photographed by a GE Amersham Typhoon imager.

### 2.12. Enzyme-Linked Immune-Sorbent Assay (ELISA)

The TGF-*β*1 secretion from mouse CFs was measured by a mouse TGF-*β*1 sandwich ELISA kit (Proteintech, KE10005). Briefly, the mouse CFs were seeded in equal numbers and received different treatment; then, they were serum starved O/N, washed, and incubated with fresh serum free medium for additional 24 hours before the supernatants were sampled by centrifugation and applied to ELISAs, which were performed all according to the instructions of the manufacturers. Specifically, the secreted TGF-*β*1 protein in the collected supernatant was pretreated by 1 N hydrochloric acid solution to become bioactive and detectable and followed by the treatment with 1.2 N sodium hydroxide/HEPES solution to be neutralized and ready for ELISAs.

### 2.13. Statistical Analysis

The data presented were collected from three independent, parallel experiments and are presented as the mean ± SEM. Statistical analysis was conducted using unpaired Student's *t* tests and one-way ANOVA (Tukey's test) with GraphPad Prism software V7.0. *p* values < 0.05 were considered to be statistically significant.

## 3. Results

### 3.1. LBH Was Upregulated in Mouse CMs and CFs during Post-MI Infarct Healing

**Figure 1:**
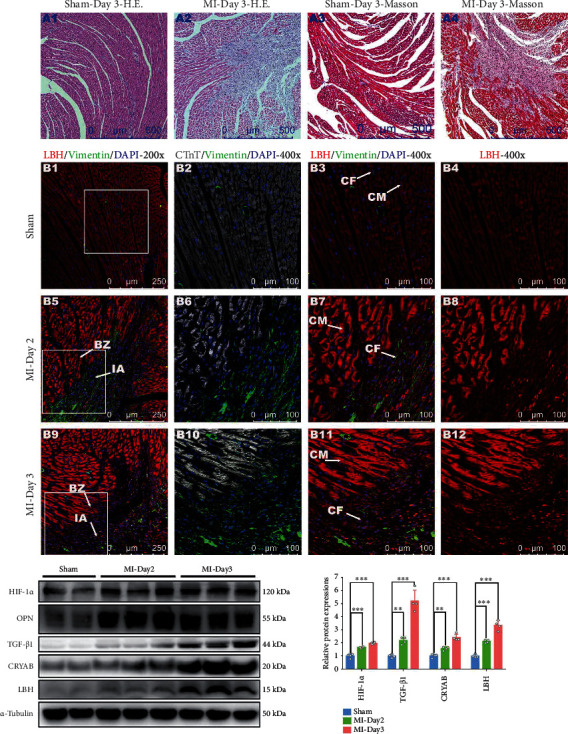
Protein expression of LBH, CRYAB, and TGF-*β*1 in heart tissue from the mouse MI model during post-MI infarct healing. (a) Cross sections of hearts from the sham and MI groups at the indicated time points after surgery were subjected to H&E and Masson staining. (b) The same heart sections were costained with anti-LBH (red) and anti-vimentin (green) or with anti-LBH (red) and anti-CTnT (gray) antibodies. Typical CFs (vimentin^+^/CTnT^−^), CMs (vimentin^−^/CTnT^+^), infarct areas (IAs), and infarct border zones (BZs) were indicated by white arrows. (c) Protein expression levels of HIF-1*α*, OPN, TGF-*β*1, LBH, and CRYAB in the peri-infarct heart tissue at the indicated time points after surgery (^∗∗^*p* < 0.01 and ^∗∗∗^*p* < 0.001 vs. Sham group).

In our previous study, we confirmed that LBH upregulation in activated CFs accumulated in the peri-infarct areas of a mouse MI model [13]. Here, we investigated the expressional changes of the LBH gene in cardiac tissue during the inflammatory phase of post-MI infarct healing. Necrotic and inflammatory areas in the MI groups were visualized after H&E staining, and fibrotic areas were observed after Masson staining, all together verifying the development of the surgery-induced MI model (Figure 1(a)). Costaining with anti-LBH and anti-vimentin, as well as anti-LBH and anti-CTnT, helped us to distinguish CFs (vimentin^+^/CTnT^−^) and CMs (vimentin^−^/CTnT^+^), and CFs were observed to be scattered in the cardiac interstitium in the sham group (Figure 1(b1–b4)), which agreed with the cardiac distribution of resident CFs [3]. In the MI groups, increased numbers of CFs were found in the infarction and peri-infarction areas on days 2 and 3 after MI (Figure 1(b5 and b9)); augmented levels of LBH proteins were observed in these CFs and in CMs within peri-infarction areas (Figure 1(b5–b12)); the expression level of LBH in CMs was significantly higher than that in CFs. In addition, western blotting of peri-infarction heart tissue also verified the post-MI LBH upregulation. This LBH upregulation was associated with elevated expression of HIF-1*α*, osteopontin (OPN), CRYAB, and TGF-*β*1 (Figure 1(c)), which was consistent with the findings reported by Jiang et al. [29] and our previous study [13]. Conclusively, during the inflammatory phase of post-MI infarct healing, enhanced protein levels of LBH were observed in both CMs and accumulated CFs in the peri-infarction areas, among which CMs exhibited relatively higher LBH expression, while upregulated LBH and CRYAB in CFs were considered to participate in the activation of CFs [13].

### 3.2. LBH Upregulation in CMs under Hypoxia Increased the Exosomal Distribution of LBH Protein in CM-Derived Exosomes

**Figure 2:**
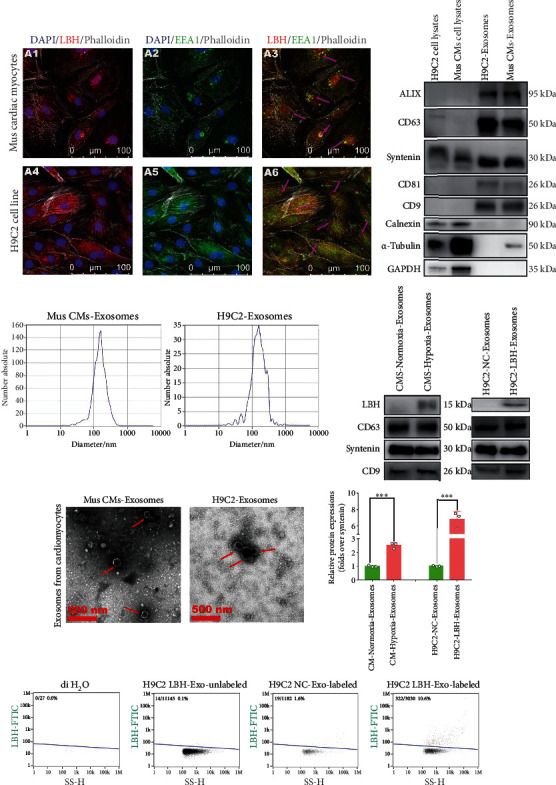
The exosomal distribution of LBH protein is elevated in the exosomes secreted by LBH-upregulated CMs under hypoxia. (a) Representative confocal microscopy images of dual staining with anti-LBH (red) and anti-EEA1 (green) in mouse primary CMs and H9c2 cells. Colocalization of LBH and EEA1 was observed in the perinuclear cytoplasm of both primary CMs and H9c2 cells (indicated by magenta arrows). (b) The distribution of particle size/concentration of exosome samples analyzed by NTA. (c) Representative TEM images of exosome samples derived from mouse primary CMs and H9c2 cells (exosomal structures were indicated by red arrows). (d) Protein expression of CD9, CD63, CD81, ALIX, syntenin-1, and calnexin in cell lysates and the corresponding CM-derived exosomes from both mouse primary CMs and H9c2 cells. (e) Western blotting measuring LBH protein levels in CM^LBH+^ exosomes and CM^NC^ exosomes (^∗∗∗^*p* < 0.001 vs. mouse CM-normoxia-exosomes/H9c2-NC-exosomes). (f) Exosome samples derived from mouse primary CMs under normoxia or hypoxia were tested by nanoflow cytometry after being stained with anti-LBH-FITC.

In both mouse CMs and H9c2 cells, LBH expression was elevated under hypoxia (Figure S5) and was higher than that in activated CFs observed in hypoxic peri-infarct areas. Additionally, LBH colocalized with the vesicle marker early endosome antigen 1 (EEA1) in mouse CMs and H9c2 cells (Figure 2(a)), suggesting possible secretion of LBH via CM exosomes. We assumed that the elevated LBH level in CFs and LBH-mediated activation of CFs in peri-infarct areas might be affected by crosstalk between these CFs and LBH-upregulated CMs and intercellular transport via exosomes might be involved in this process. Therefore, exosomes derived from both mouse CMs and H9c2 cells were isolated and identified to test this assumption. First, the average sizes of the major components of isolated samples were around 150 nm as measured by NTA (Figure 2(b)); then, clear lipid bilayer membranes with major diameters of 100-200 nm were observed in transmission electron microscopy (TEM) images (Figure 2(c)). Finally, the isolated samples exhibited higher expression of the exosomal markers CD9, CD63, CD81, ALIX, and syntenin-1, as well as lower expression of housekeeping genes than the corresponding cell lysates (Figure 2(d)). Moreover, western blotting and nanoflow cytometry uniformly confirmed that hypoxic CM-derived exosomes showed a higher LBH distribution than normoxic CM-derived exosomes, and similar results were found for H9c2-LBH-derived exosomes compared to H9c2-NC-derived exosomes (Figures 2(e) and 2(f)). These results indicated that LBH protein was secreted by CMs via exosomes and that hypoxia-induced LBH upregulation in CMs led to enhanced exosomal distribution of LBH protein.

### 3.3. The Delivery of LBH Protein from CMs to CFs Was Mediated by CM-Derived Exosome Secretion

**Figure 3:**
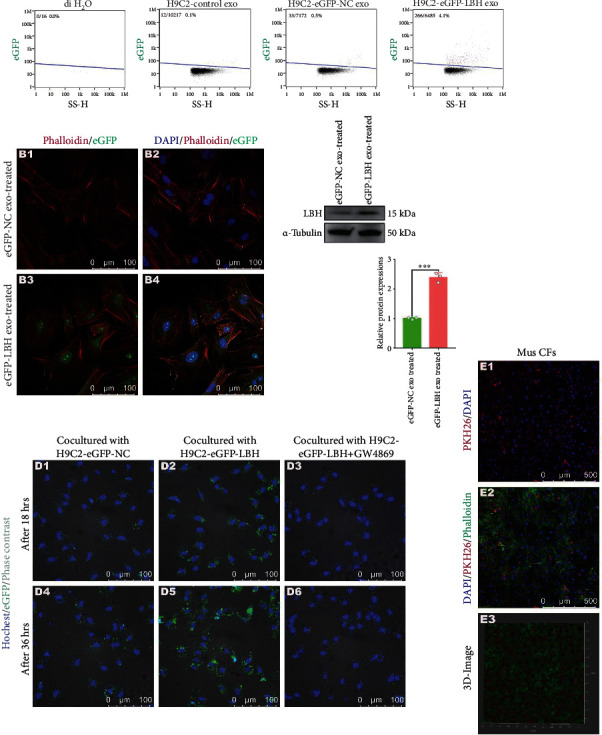
Cellular uptake of LBH protein from CM-derived exosomes into CFs. (a) LBH-eGFP fusion protein was detected in exosome samples derived from LBH-eGFP plasmid-transfected H9c2 cells by nanoflow cytometry. (b) Representative confocal microscopic images of rat CFs treated with LBH-eGFP exosomes. (c) Protein levels of LBH in rat CFs treated with LBH-eGFP exosomes or NC exosomes (^∗∗∗^*p* < 0.001 vs. CFs treated with H9c2-eGFP-NC exosomes). (d) Representative confocal microscopic images of rat CFs cocultured with LBH-eGFP plasmid-transfected H9c2 cells with or without the exosome production inhibitor GW4869. (e) Representative confocal microscopic images of mouse CFs treated with CM exosomes labeled with a PKH26 kit.

Since the secretion of LBH protein from CMs was shown to be implemented by CM exosomes, we further explored whether these exosomal LBH proteins could be taken up by CFs. After H9c2 cells were transfected with eGFP-NC/eGFP-LBH plasmids and screened by antibiotics, their culture media were collected and ultracentrifuged for exosome isolation. Nanoflow cytometry detection of the isolated samples showed higher eGFP^+^ rates in H9c2-eGFP-LBH derived exosomes than in the negative controls, indicating considerable distribution of eGFP-LBH fusion proteins in exosomes (Figure 3(a)). Then, rat CFs were treated with H9c2-eGFP-LBH derived exosomes and showed marked intracellular eGFP signals, which manifested successful cellular uptake of LBH proteins via exosome internalization (Figure 3(b)). Also, the exosome treated rat CFs were subjected to western blotting, and the CFs treated with LBH-eGFP exosomes showed augmented LBH protein levels compared to the negative controls (Figure 3(c)). Similarly, when rat CFs were cocultured with LBH-eGFP plasmid-transfected H9c2 cells, eGFP signals were detected inside CFs, which implied the intercellular delivery of LBH protein from H9c2 cells to CFs (Figure 3(d)), and these H9c2 cells did not migrate through the insert membranes during the coculture (Figure S9). Treatment of H9c2-LBH-eGFP cells with exosome production inhibitor caused decreased eGFP signals detected inside the CFs cocultured with treated H9c2-LBH-eGFP cells, suggesting that exosome production, secretion, and internalization participated in the intercellular delivery of LBH protein from H9c2 cells to CFs (Figure 3(d)). Additionally, mouse CFs were treated with mouse CM-derived exosomes labeled with PKH26 and showed significant cellular uptake of the labeled exosomes (Figure 3(e)). These data suggested that in the cardiac microenvironment, CM-originated exosomes could be internalized by adjacent CFs, and since LBH upregulation under hypoxia led to enhanced LBH packaging in CM exosomes, the cellular uptake of these LBH-enriched (LBH^+^) exosomes could increase the LBH protein level in CFs that act as recipient cells.

### 3.4. LBH+ Exosomes Secreted by LBH-Overexpressing CMs Promoted the Proliferation, Differentiation and Migration of CFs *In Vitro*

**Figure 4:**
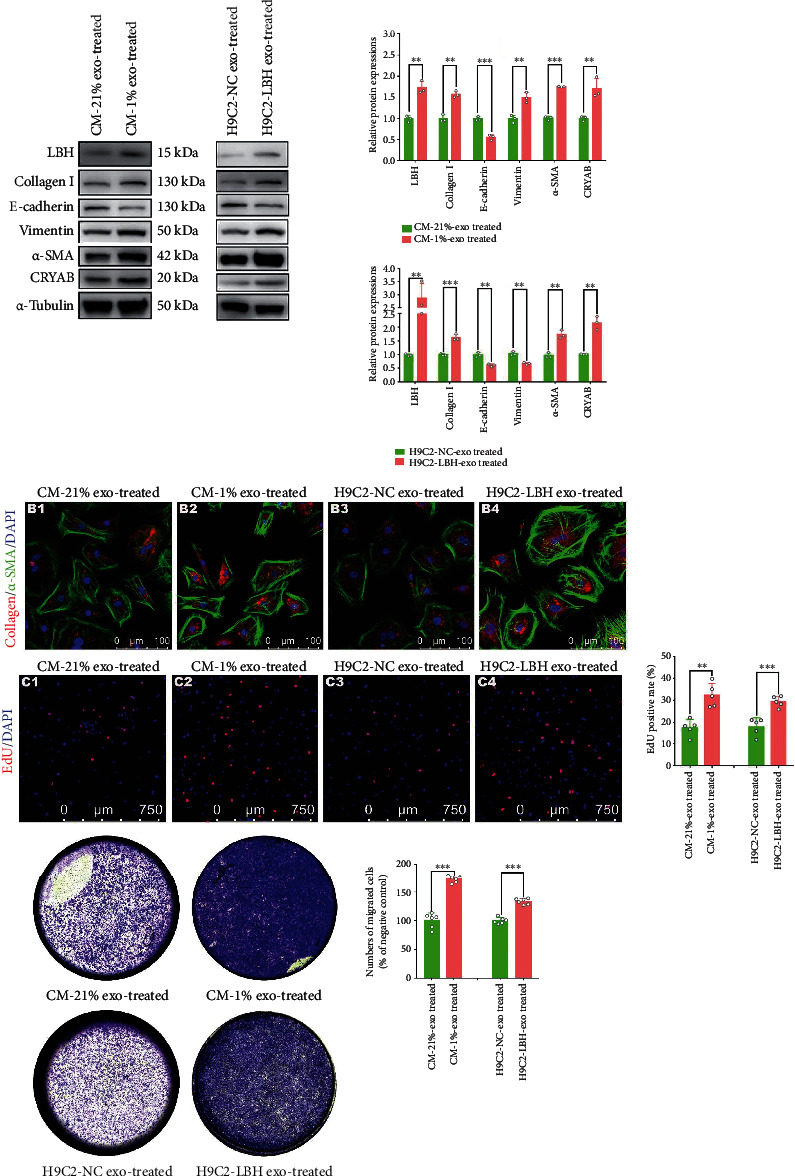
The effects of the internalization of CM^LBH+^ exosomes on the proliferation, differentiation and migration of CFs. (a) Protein levels of LBH, CRYAB, collagen I, *α*-SMA, E-cadherin, and vimentin in mouse CFs treated with hypoxic CM-derived exosomes and in rat CFs treated with H9c2-LBH-derived exosomes (^∗∗^*p* < 0.01 and ^∗∗∗^*p* < 0.001 vs. 21%-Exo/Lv5NC-Exo treated). (b) Representative immunofluorescence images of *α*-SMA and collagen I expression in mouse CFs treated with hypoxic CM-derived exosomes and in rat CFs treated with H9c2-LBH-derived exosomes. (c) EdU staining of mouse CFs treated with hypoxic CM-derived exosomes and rat CFs treated with H9c2-LBH-derived exosomes (^∗∗^*p* < 0.01 and ^∗∗∗^*p* < 0.001 vs. 21%-Exo/Lv5NC-Exo treated). (d) Representative images of the Transwell assay of mouse CFs treated with hypoxic CM-derived exosomes and rat CFs treated with H9c2-LBH-derived exosomes and the corresponding statistical analysis (^∗∗∗^*p* < 0.001 vs. 21%-Exo/Lv5NC-Exo treated).

Since the increased level of LBH protein in CFs could be achieved by the internalization of LBH^+^ exosomes, while LBH was reported to be involved in CF activation according to our previous research [13], assays testing various phenotypes related to CF activation were conducted *in vitro*. Treatment with both hypoxic mouse CM-derived exosomes and H9c2-LBH-derived exosomes caused the upregulation of LBH and CRYAB in mouse CFs and rat CFs, respectively, compared to CFs cocultured with normoxia/NC exosomes, together with elevated expression of collagen I, *α*-SMA, vimentin, and diminished E-cadherin expression (Figures 4(a) and 4(b)), which indicated promoted LBH-induced transdifferentiation and EMT-like processes of CFs. Correspondingly, both hypoxic mouse CM-derived exosomes and H9c2-LBH-derived exosomes accelerated the proliferation (Figures 4(c) and S10) and migration (Figure 4(d)) of mouse/rat CFs compared to the negative controls, which is in accordance with their promoted FMT progression. Therefore, we concluded that LBH+ exosomes secreted by LBH-overexpressing CMs facilitated the upregulation of LBH and CRYAB in CFs, which were verified to promote the proliferation, differentiation, and migration of CFs.

### 3.5. LBH Knockout Partially Inhibited TGF-*β*1-Induced CF Activation

**Figure 5:**
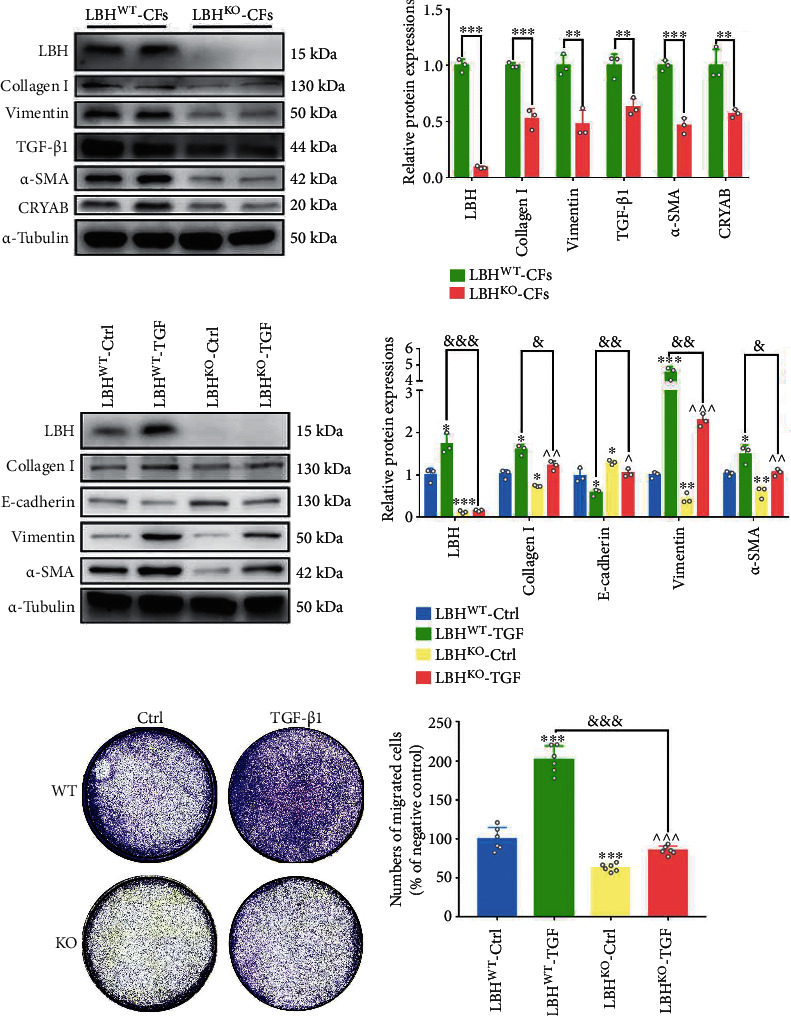
CFs isolated from LBH^KO^ mice showed partially mitigated CF activation under TGF-*β* stimulation. (a) Protein levels of LBH, CRYAB, collagen I, *α*-SMA, E-cadherin, vimentin, and TGF-*β*1 in CFs isolated from LBH^WT^ and LBH^KO^ mice (^∗∗^*p* < 0.01 and ^∗∗∗^*p* < 0.001 vs. LBH^WT^). (b) Protein levels of LBH, collagen I, *α*-SMA, E-cadherin, and vimentin in both LBH^WT^ and LBH^KO^ mouse CFs under TGF-*β* stimulation (^∗^*p* < 0.05, ^∗∗^*p* < 0.01, and ^∗∗∗^*p* < 0.001 vs. LBH^WT^-Ctrl; ∧*p* < 0.05, ∧∧*p* < 0.01, and ∧∧∧*p* < 0.001 vs. LBH^KO^-Ctrl; ^&^*p* < 0.05, ^&&^*p* < 0.01, and ^&&&^*p* < 0.001 vs. LBH^WT^-TGF). (c) Representative images of the Transwell assay of LBH^WT^ and LBH^KO^ mouse CFs under TGF-*β* stimulation and the corresponding statistical analysis (^∗∗∗^*p* < 0.001 vs. LBH^WT^-Ctrl; ∧∧∧*p* < 0.001 vs. LBH^KO^-Ctrl; ^&&&^*p* < 0.001 vs. LBH^WT^-TGF).

The roles of the LBH gene in CF activation were verified by introducing both overexpression and knockdown lentiviruses in our previous study. Here, to further investigate the LBH-related mechanisms during this process, primary CFs were isolated from established LBH^KO^ mice and LBH^WT^ mice. According to the WB results, LBH knockout caused inhibited protein expression of CRYAB, collagen I, *α*-SMA, vimentin, and TGF-*β*1, as well as increased E-cadherin expression in mouse CFs (Figures 5(a) and 5(b)); TGF-*β*1 stimulation upregulated protein expression of LBH, collagen I, *α*-SMA, vimentin, and mitigated E-cadherin expression in LBH^WT^ CFs, and these effects were partially reversed in LBH knockout groups (Figure 5(b)). Besides, although LBH expression was knocked out, TGF-*β*1 stimulation still elevated the expression of collagen I, *α*-SMA, and vimentin in LBH^KO^ CFs (Figure 5(b)). Consistent with the protein expressional changes, TGF-*β*1 stimulation promoted the migration of both LBH^KO^ and LBH^WT^ CFs, while LBH knockout attenuated CF proliferation and migration with or without TGF-*β*1 treatment (Figures 5(c) and S11). Based on the results, we believed that LBH-CRYAB signaling participated in TGF-*β*1-induced CF activation; specifically, LBH was indispensable in part of the effectuation of TGF-*β*1-induced transdifferentiation and EMT-like processes, while part of TGF-*β*1-induced CF activation was independent of LBH mediation.

### 3.6. LBH Affected TGF-*β*1 Expression and Signaling through a Positive Feedback Loop

**Figure 6:**
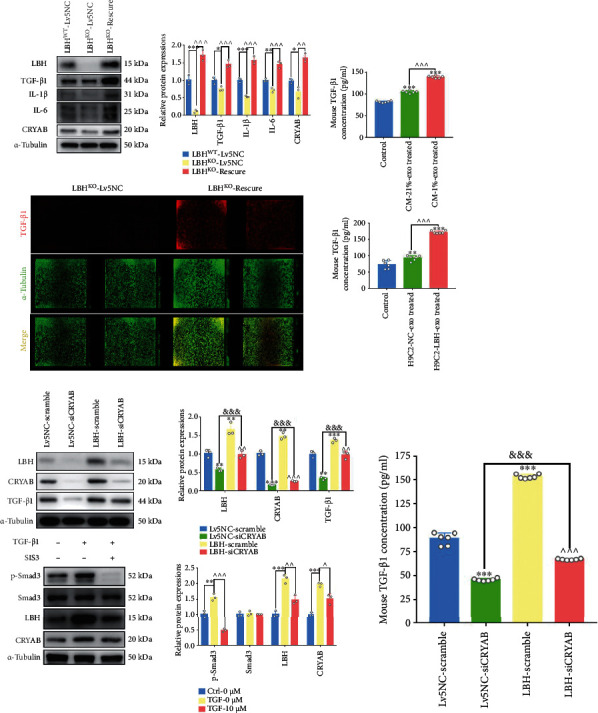
LBH affects TGF-*β*1 expression and signaling through a positive feedback loop. (a) Protein levels of LBH, CRYAB, TGF-*β*1, IL-1*β*, and IL-6 in LBH^KO^ mouse CFs rescued by lentiviral-induced LBH upregulation (^∗^*p* < 0.05, ^∗∗^*p* < 0.01, and ^∗∗∗^*p* < 0.001 vs. LBH^WT^-Lv5NC; ∧∧*p* < 0.01 and ∧∧∧*p* < 0.001 vs. LBH^KO^- Lv5NC). (b) In cell western simultaneously testing, the protein levels of TGF-*β*1 (red) and *α*-tubulin (green) in LBH^KO^ mouse CFs transfected with Lv5NC or LBH lentivirus. (c) Secreted TGF-*β*1 in the cell supernatants of mouse CFs treated with LBH+ exosomes, together with LBH^KO^ CFs rescued by LBH-overexpressing lentivirus, were measured by ELISAs (^∗∗∗^*p* < 0.001 vs. Control/LBH^WT^-LvNC; ∧∧∧*p* < 0.001 vs. CM-21%-Exo-treated/LBH^KO^-Lv5NC). Protein expression of LBH, CRYAB, TGF-*β*1 (d), and TGF-*β*1 secretion (e) in mouse CFs after LBH overexpression and subsequent CRYAB knockdown (^∗∗^*p* < 0.01 and ^∗∗∗^*p* < 0.001 vs. Lv5-NC-Scramble; ^&&&^*p* < 0.001 for Lv5-NC-siCRYAB vs. LBH-siCRYAB; ∧∧p < 0.01 and ∧∧∧*p* < 0.001 for LBH-Scramble vs. LBH-siCRYAB). (f) Protein levels of LBH, p-Smad3 and Smad3 in TGF-*β*1-stimulated mouse CFs with or without treatment with the phosphorylation inhibitor SIS3 (^∗∗^*p* < 0.01 and ^∗∗∗^*p* < 0.001 vs. Ctrl-0 *μ*M SIS3; ∧*p* < 0.05, ∧∧*p* < 0.01, and ∧∧∧*p* < 0.001 vs. TGF-0 *μ*M SIS3).

In our previous study, we demonstrated the role of CRYAB in mediating enhanced TGF-*β*1 expression in CFs through a positive feedback loop [13], which was also in line with Bellaye et al. [30]. Since LBH-induced CRYAB upregulation in CFs had been previously validated, we speculated that LBH might affect TGF-*β*1 expression through similar mechanisms. Thus, a knockout-rescue system of the LBH gene was established by introducing LBH-overexpressing lentivirus into LBH^KO^ CFs, and WB results indicated that the re-expression of LBH in LBH^KO^ CFs increased the protein levels of CRYAB, TGF-*β*1, IL-1*β*, and IL-6 (Figures 6(a) and 6(b)). Since the mediation of TGF-*β* signaling requires extracellular, free TGF-*β* ligands to bind and activate membrane-located TGF-*β* receptors, we also detected TGF-*β*1 levels in culture supernatants by ELISAs. The data indicated that in mouse CFs, increased extracellular TGF-*β*1 levels could be induced by hypoxic mouse CM-derived exosomes (Figure 6(c)), which corresponded to upregulated LBH expression in CFs treated with LBH+ exosomes (Figure 4(a)); LBH knockout decreased extracellular TGF-*β*1 levels, which could be restored by the re-expression of LBH in LBH^KO^ CFs (Figure 6(c)), and these results were also in accordance with the expression of LBH and TGF-*β*1 in CFs (Figures 6(a) and 6(b)). Moreover, when we transfected siCRYAB oligos into mouse CFs that had previously been infected with Lv5-NC or Lv5-LBH lentivirus, the WB results indicated that LBH overexpression elevated the expression of CRYAB and TGF-*β*1, as well as TGF-*β*1 secretion in both scramble-transfected and siCRYAB-transfected CFs, while CRYAB knockdown downregulated LBH and TGF-*β*1 expression, as well as TGF-*β*1 secretion in both negative controls and LBH-overexpressing CFs (Figures 6(d) and 6(e)). Additionally, after administering the Smad3 phosphorylation inhibitor SIS3 to CFs under TGF-*β*1 stimulation, we found that suppressing Smad3 phosphorylation partially reversed the TGF-*β*1-induced upregulation of LBH and CRYAB (Figure 6(f)). Altogether, we concluded that LBH is related to the expression of certain inflammatory factors during the inflammatory phase of post-MI infarct healing; also, LBH promotes TGF-*β*1 expression, secretion, and signaling through a positive LBH-CRYAB-TGF *β*1 feedback loop, while the canonical TGF-*β*-Smad2/3 pathways are involved in TGF-*β*1-induced.

## 4. Discussion

The LBH gene is specifically expressed in the embryonic limb and heart and has important roles during normal cardiogenesis [31]. In our previous study, post-MI LBH upregulation was detected in the infarct border zone and was responsible for CF activation and accumulation, thus participating in post-MI infarct healing [13]. Specifically, we found that hypoxia, as indicated by HIF-1*α* expression, only occurred in the infarct border zone during the inflammatory phase of post-MI infarct healing, together with LBH upregulation. After peaking, LBH expression starts to decline during the maturation phase (Figure S12), when physoxia [32] was restored in injured cardiac tissues, and this change was synchronized with diminished HIF-1*α* expression. HIF-1*α* activation has been reported to transcriptionally regulate LBH expression by directly binding its upstream promoter [29]; therefore, we focused on the effects and mechanisms of the LBH gene on cardiac repair under hypoxic conditions. Among the results of our preliminary experiments, the elevated HIF-1*α* levels on days 2 and 3 post-MI verified the hypoxic cardiac microenvironment, while the upregulation of TGF-*β*1 and OPN1 proved the occurrence of inflammatory reactions and cardiac repair [33, 34] and was consistent with Dewald et al. [35] and Deten et al. [36]. In addition, it is noteworthy that the upregulation of LBH and CRYAB was observed in both CMs and CFs in the infarct border zone during this inflammatory phase. These synchronous expression changes raised the question that whether the previously proven LBH-triggered CF activation is influenced by LBH upregulation in hypoxic CMs in the same cardiac microenvironment, which was further explored in this study.

Recent studies have revealed the relevance between intercellular communication mediated by CM-derived exosomes and the pathogenesis of various heart diseases, including acute and chronic cardiac fibrosis [37–40]. In both mouse primary CMs and H9c2 cells used in this study, LBH proteins colocalized with EEA1 proteins, implying its distribution in internal vesicles and possible extracellular release via CM exosome secretion. Besides, in various studies, the upregulation of stress-induced proteins in CMs led to elevated exosomal distribution [17]. For our CM-derived exosomes, the samples isolated from LBH-upregulated CMs under hypoxia exhibited relatively higher exosomal LBH protein levels. Since the LBH level in CMs in the infarct border zone was considerably higher than that in accumulated CFs (Figure 1(b)), we supposed that the increased LBH proteins observed in CFs might partially originate from CMs in the same post-MI cardiac microenvironment and might be delivered into CFs by CM exosomes. To examine the process of intercellular communication, a labeling and tracking scheme of the LBH protein was designed by introducing an LBH-eGFP fusion protein expression vector. The LBH-eGFP expressed in CMs was secreted into CM-derived exosomes and taken up by CFs, resulting in augmented LBH levels in recipient CFs, which also agreed with our coculture results by introducing exosome production and secretion inhibitor (Figure S13) [41, 42]. Since we have already demonstrated the modulation of upregulated LBH expression on CF proliferation, transdifferentiation, and migration [13], the possible effects of LBH^+^ CM exosomes on CFs require our further verification.

Then, the correlated phenotypes of CFs treated with LBH^+^ CM exosomes (both hypoxic CM-derived exosomes and H9c2-LBH-derived exosomes) were examined. The fact that hypoxic CM-derived exosome-treated CFs and H9c2-LBH-derived exosome-treated CFs uniformly showed enhanced LBH levels, along with promoted FMT, proliferation, mesenchymal characteristics (Figure S14) and cellular migration confirmed our presumption that LBH^+^ CM exosomes could trigger CF activation upon internalization by upregulating LBH levels in CFs. Since the purity of primary CFs used in this study had been verified (Figure S3), the advanced mesenchymal characteristics and mitigated epithelial characteristics of MCFs indicated by the WB results should be attributed to the “EMT-like process” of CFs reported by some researchers [43, 44], rather than endothelial-mesenchymal transition (EndMT) of endothelial cells post-MI [45, 46]. Besides, in our previous study, the accelerated proliferation of LBH-overexpressing CFs was dependent on hypoxia status [13]; here, not only did the attenuated proliferation of LBH^KO^ CFs validate the proliferative regulation of the LBH gene in CFs, but the H9c2-LBH-derived exosome-treated CFs also exhibited accelerated proliferation without hypoxic conditions, which might be due to certain exosomal components and needs to be explored in the future.

The regulation of CF activation by LBH, as described above, was also reversely confirmed in LBH^KO^ CFs, which showed inhibited transdifferentiation, EMT-like processes, and cellular migration. In our previous researches, the regulatory effects of the LBH gene as a transcriptional cofactor were confirmed to be induced by TGF-*β*1 stimulation. After introducing TGF-*β*1 stimulation into LBH^KO^ CFs, we found that LBH^KO^ CFs treated with TGF-*β*1 showed hampered corresponding phenotypes compared to TGF-*β*1 treated LBH^WT^ CFs and promoted phenotypes compared to that of untreated LBH^KO^ CFs. That is, TGF-*β*1-induced CF activation partially relies on the mediation of LBH, while the other part is irrelevant to TGF-*β*1-induced LBH signaling. Additionally, because hypoxia-invoked HIF-1*α* activation transcriptionally regulates LBH expression [29], we hypothesize that the ascending LBH level observed in CFs in the post-MI cardiac microenvironment might be partially independent of TGF-*β*1-related induction during the inflammatory phase, when LBH proteins could be transferred from LBH-upregulated hypoxic CMs as we proved. The testification of our hypothesis, however, requires the introduction of specific inhibitors of the TGF-*β* receptor [47, 48] or TGF-*β* neutralizing antibodies [49], both of which currently have limitations and could be our next research topic.

As the predominant mediator of fibrogenesis, TGF-*β* has been extensively studied, and its signaling during reactive and replacement cardiac fibrosis is generally defined into two categories: the canonical TGF-*β*/Smad pathway and the noncanonical TGF-*β* pathway without Smad family members [50, 51]. In this study, the inhibition of Smad3 phosphorylation partially reversed the TGF-*β*1-induced LBH upregulation in CFs, indicating the involvement of TGF-*β*/Smad signaling. Moreover, the mechanism of LBH-mediated CF activation under TGF-*β*1 stimulation was proven to be correlated with CRYAB upregulation in our previous research [13]; here, the WB and ELISA results of LBH-rescued LBH^KO^ CFs bidirectionally verified that TGF-*β*1 expression and secretion were positively regulated by LBH in CFs. Since CRYAB has been reported to regulate TGF-*β*1 expression and signaling in pleural fibrosis through an autoinduction loop [52], we hypothesized that CRYAB might affect LBH-regulated TGF-*β*1 expression in CFs through a positive feedback loop. Based on the result that introducing siCRYAB into LBH-overexpressing CFs downregulated the expression and secretion of TGF-*β*1, we concluded that CRYAB did mediate the positive feedback regulation of TGF-*β*1, which in turn activated TGF-*β*1-mediated LBH-CRYAB signaling. Thus, LBH supplementation via CM exosomes could promote TGF-*β*1 signaling in CFs through this feedback loop, leading to enhanced TGF-*β*1-mediated CF activation during the inflammatory phase. Moreover, CM-derived exosomes have been reported to specifically participate in inflammatory regulation [19]. In summary, we propose that LBH+ CM exosomes may serve as potential adjuvant treatment for post-MI injuries.

## 5. Conclusion

**Figure 7:**
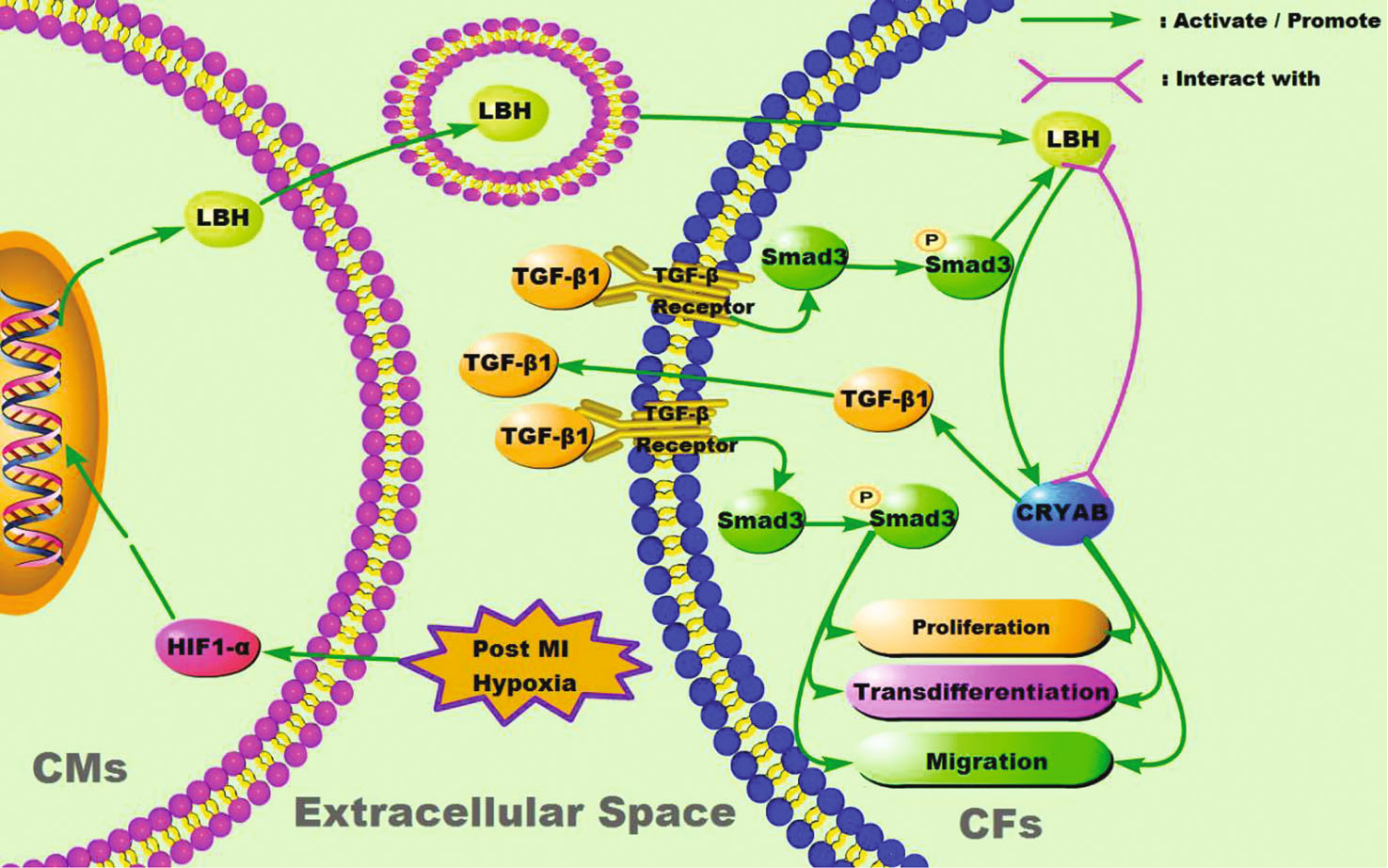
Schematic summary of exosomal LBH related signaling in the post-MI cardiac microenvironment. The LBH-CRYAB signaling responsible for modulating the proliferation, transdifferentiation, and migration of CFs could be induced by exosome secretion from LBH-upregulated CMs under hypoxia.

During the inflammatory phase of cardiac fibrosis, LBH expression was elevated in hypoxic CMs in the infarct border zone, and upregulated LBH protein could be transferred into CM exosomes and internalized by nearby CFs, together with the increased TGF-*β*1 level in the cardiac microenvironment, jointly causing LBH upregulation in CFs. This upregulation promoted the LBH-modulated proliferative acceleration, transdifferentiation, and EMT-like process of CFs, which contributed to post-MI infarct healing implemented by activated CFs (Figure 7). Since the CM-exosome-mediated LBH upregulation in CFs might also promote TGF-*β*1-mediated CF activation through the LBH-CRYAB-TGF *β*1 feedback loop, these findings provide new insights into the potential therapeutic applications of LBH in cardiac repair and anti-fibrotic therapy. More detailed and direct evidence, however, is still needed to validate and explore our hypothesis.

## Data Availability

All the data of this manuscript is available on honorable request to corresponding authors.

## Supplementary Materials

Supplementary Materials Supplemental Figure 1. The establishment of LBH conventional knockout (KO) C57BL/6 mice and corresponding verification. Supplemental Figure 2. H.E. staining, Masson staining, and anti-LBH staining of the whole heart slides in C57 mice model of MI. Supplemental Figure 3. Immunofluorescence images of primary mouse CMs and H9c2 cell lines for the staining of anti-cardiac myosin, anti-alpha actinin (ATCN1), and anti-cardiac troponin t (CTnT), as well as immunofluorescence images of primary mouse CFs and rat CFs for the staining of anti-vimentin, anti-collagen I, anti-*α*-SMA, anti-PDGFR1, and anti-S100A4. Supplemental Figure 4. Immunofluorescence images indicating the infection/transfection efficiencies of puromycin-screened H9c2 cell lines prepared for the following experiments. Supplemental Figure 5. Representative images of H9c2 cells and mouse CMs for phase contrast imaging and western blotting detecting protein levels of LBH and HIF-1*α* in H9C2 cells and mouse CMs under hypoxia (1% O_2_). Supplemental Figure 6. Protocols of exosome isolation by differential ultracentrifugation used in this study, presented as flowchart. Supplemental Figure 7. The time length gradient and concentration gradient tests for mouse CFs treated with Smad3 phosphorylation inhibitor SIS3. Supplemental Figure 8. The concentration gradient test for H9c2 cells treated with exosome secretion inhibitor GW4869, which was used for coculture process. Supplemental Figure 9. The crystal violet staining of transwell inserts used for coculture to exclude cell contact between H9c2 cells and rat CFs during the coculture process. Supplemental Figure 10. The effects of CM-derived exosomes on CFs proliferation indicated by EdU staining were verified by CCK-8 assay and anti-Ki67 staining. Supplemental Figure 11. The effects of LBH gene on CFs proliferation and migration were verified by LBH knockout, indicated by EdU staining and transwell assay, respectively. Supplemental Figure 12. Protein expression levels of LBH and CRYAB of peri-infarction heart tissue at the indicated time points during chronic cardiac fibrosis induced by the coronary artery ligation. Supplemental Figure 13. Protein expression levels of LBH in LBH^WT^ CFs cocultured with LBH^WT^/LBH^KO^ CMs under the treatment with inhibitor GW4869. Supplemental Figure 14. Representative immunofluorescence images of vimentin and E-cadherin expression in mouse CFs treated with hypoxic CM-derived exosomes and in rat CFs treated with H9C2-LBH-derived exosomes. Supplemental Table 1. Antibodies for Western blotting and Immunofluorescence.
