# Supplementary material for: Limb-Bud and Heart (LBH) Upregulation in Cardiomyocytes under Hypoxia Promotes the Activation of Cardiac Fibroblasts via Exosome Secretion
Source: Mediators Inflamm. 2022 Sep 6;2022:8939449. doi: 10.1155/2022/8939449 (PMC9470350; doi:10.1155/2022/8939449)
Supplement: Supplementary Materials — Supplemental Figure 1. The establishment of LBH conventional knockout (KO) C57BL/6 mice and corresponding verification. Supplemental Figure 2. H.E. staining, Masson staining, and anti-LBH staining of the whole heart slides in C57 mice model of MI. Supplemental Figure 3. Immunofluorescence images of primary mouse CMs and H9c2 cell lines for the staining of anti-cardiac myosin, anti-alpha actinin (ATCN1), and anti-cardiac troponin t (CTnT), as well as immunofluorescence images of primary mouse CFs and rat CFs for the staining of anti-vimentin, anti-collagen I, anti-α-SMA, anti-PDGFR1, and anti-S100A4. Supplemental Figure 4. Immunofluorescence images indicating the infection/transfection efficiencies of puromycin-screened H9c2 cell lines prepared for the following experiments. Supplemental Figure 5. Representative images of H9c2 cells and mouse CMs for phase contrast imaging and western blotting detecting protein levels of LBH and HIF-1α in H9C2 cells and mouse CMs under hypoxia (1% O2). Supplemental Figure 6. Protocols of exosome isolation by differential ultracentrifugation used in this study, presented as flowchart. Supplemental Figure 7. The time length gradient and concentration gradient tests for mouse CFs treated with Smad3 phosphorylation inhibitor SIS3. Supplemental Figure 8. The concentration gradient test for H9c2 cells treated with exosome secretion inhibitor GW4869, which was used for coculture process. Supplemental Figure 9. The crystal violet staining of transwell inserts used for coculture to exclude cell contact between H9c2 cells and rat CFs during the coculture process. Supplemental Figure 10. The effects of CM-derived exosomes on CFs proliferation indicated by EdU staining were verified by CCK-8 assay and anti-Ki67 staining. Supplemental Figure 11. The effects of LBH gene on CFs proliferation and migration were verified by LBH knockout, indicated by EdU staining and transwell assay, respectively. Supplemental Figure 12. Protein expression levels of [file 8939449.f1.docx]

**Supplemental Table 1. Antibodies for Western blotting and Immunofluorescence**

| **Antibodies** | **Product numbers** | **Applications** |
| --- | --- | --- |
| rabbit anti-LBH | Abcam, ab173737 | WB (1:100) |
| rabbit anti-LBH | Abcam, ab122223 | IF/IHF (1:100) |
| rabbit-anti-LBH-FTIC | Lifespan LS-C672608 | NFC (1:25) |
| rabbit anti-CRYAB  rabbit anti-  phospho-CRYAB | Abcam, ab76467  Abcam, ab5577 | WB (1:500)  WB (1:2000) |
| rabbit anti-Vimentin  rat anti-Vimentin | Boster, Pb0378  R&D, Mab2105 | WB (1:400)  IF/IHF (1:200) |
| mouse anti-E-cadherin | BD, 610182 | WB (1:2000) |
| rabbit anti-α-SMA  mouse anti-α-SMA | Novus, NBP2-67440  Sigma, A5228 | WB (1:800)  IF (1:400) |
| goat anti-collagen I | Southern Biotech, 1310-01 | WB (1:800); IF (1:200) |
| rabbit anti-HIF-1α  rabbit-anti-TGF-β1  rabbit anti-IL-6  rabbit-anti-IL-β1  rabbit anti-Ki67  rabbit anti-Calnexin | Invitrogen, PA1-16601  Proteintech, 21898-1  CST, 12912  CST, 12242  Proteintech, 27309-1  Abcam, ab133615 | WB (1:800)  WB/ICW (1:800)  WB (1:800)  WB (1:800)  IF (1:100)  WB (1:800) |
| rabbit anti-CD9 | Bioss, bs-2489R | WB (1:500) |
| rabbit anti-CD63  rabbit anti-CD81  rabbit anti-ALIX  rabbit anti-Syntenin  mouse anti-EEA1  rabbit anti-PDGFR1  rabbit anti-S100A  mouse anti-CTnT  rabbit anti-α-Actinin  rabbit anti-Cardiac myosin  rabbit anti-p-Smad3  rabbit anti-Smad3 | Wanlei, WL02549  Bioss, bs-6934R  Wanlei, WL03063  Abcam, ab19903  Abcam, ab70521  Wanlei, WL02363  Proteintech, 16027-1  Invitrogen, MA5-12960  Abcam, ab68194  Abcam, ab224046  CST, 9520  CST, 9523 | WB (1:1000)  WB (1:500)  WB (1:800)  WB (1:800)  IF (1:100)  IF (1:200)  IF (1:200)  IF/IHF (1:1000)  IF (1:1000)  IF (1:2000)  WB (1:800)  WB (1:800) |
| rabbit anti-GAPDH | Bioworld, AP0063 | WB (1:8000) |
| mouse anti-α-Tubulin | Proteintech, 66031-1 | WB/ICW (1:2000) |
| goat anti-rabbit IgG-HRP | CST, 7074 | WB (1:4000) |
| horse anti-mouse IgG-HRP  donkey anti-goat IgG-HRP | CST, 7076  Invitrogen, PA1-28664 | WB (1:4000)  WB (1:4000) |
| goat anti-mouse-Alexa Fluor 488 | Invitrogen, A-11001 | IF/IHF (1:500) |
| goat anti-rabbit-Alexa Fluor Plus 555 | Invitrogen, A-21472 | IF/IHF (1:500) |
| goat anti-rat-  Alexa Fluor Plus 647  donkey anti-goat  Alexa Fluor Plus 647  Alexa Fluor Plus 680  goat anti-rabbit | Invitrogen, A-21247  Invitrogen, A-21447  Invitrogen A-21057 | IF/IHF (1:500)  IF (1:500)  IHF (1:500) |

WB, western blotting; IF, Immunofluorescence; IHF, Immunohistofluorescence; ICW, In cell western; NFC, Nano-flow cytometry.

**Supplemental Figure 1**

**
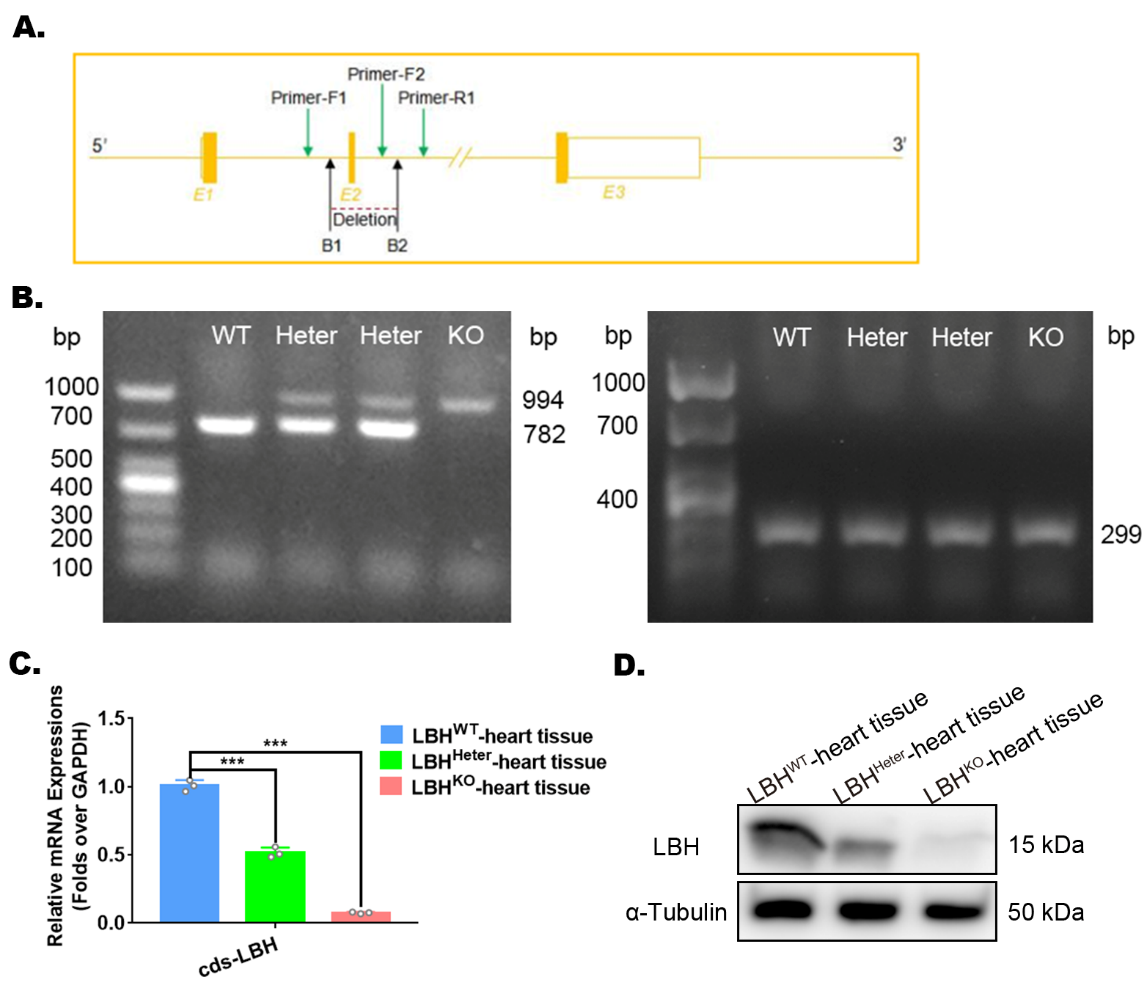
**

**Supplemental Figure 1.** The establishment of LBH conventional knockout (KO) C57BL/6 mice and corresponding verification. **(A)** Schematic representation of the mouse LBH genomic locus and designated targeting strategy used to produce and identify LBH conventional knockout mice. **(B)** RT-PCR using mouse tail genomic DNA to identify the genotypes of LBH allele. The band size 994 bp, 782 bp and 299 bp correspond to PCR product generated from primer pair F1+R1, from primer pair F2+R1, and PCR product for mouse GAPDH, respectively. QPCR **(C)** and representative image of Western blotting **(D)** detecting the LBH protein expression in heart tissue of the wildtype, heterozygote and homozygote of LBH^KO^ mice, respectively (***p<0.001 vs. LBH^WT^-heart tissue).

**Supplemental Figure 2**


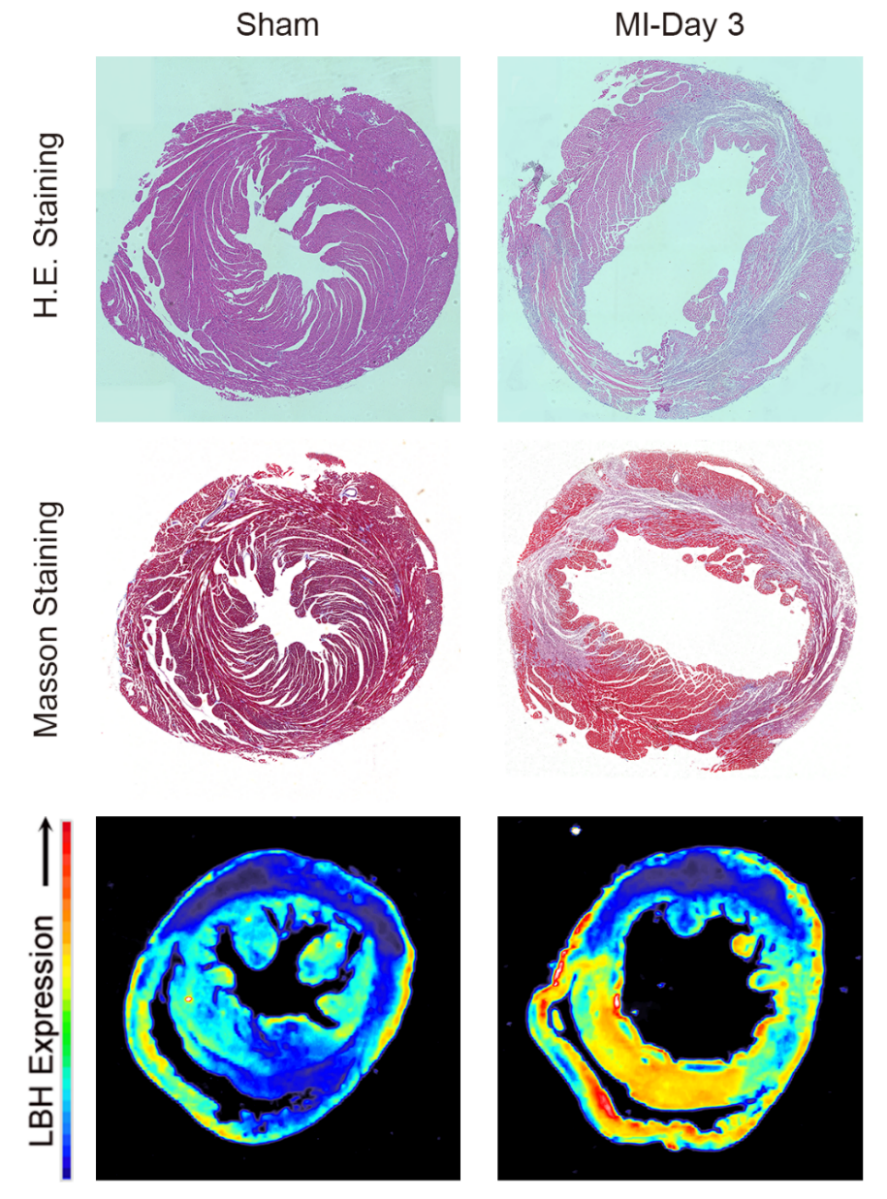


**Supplemental Figure 2.** H.E. staining, masson staining and anti-LBH staining of the whole heart slides in C57 mice model of MI. Necrotic and inflammation areas on MI slides were visualized by H.E. staining, and fibrotic areas were observed by Masson staining; elevated protein expression of LBH is observed in the necrotic and inflammation areas on MI slides.

**Supplemental Figure 3**


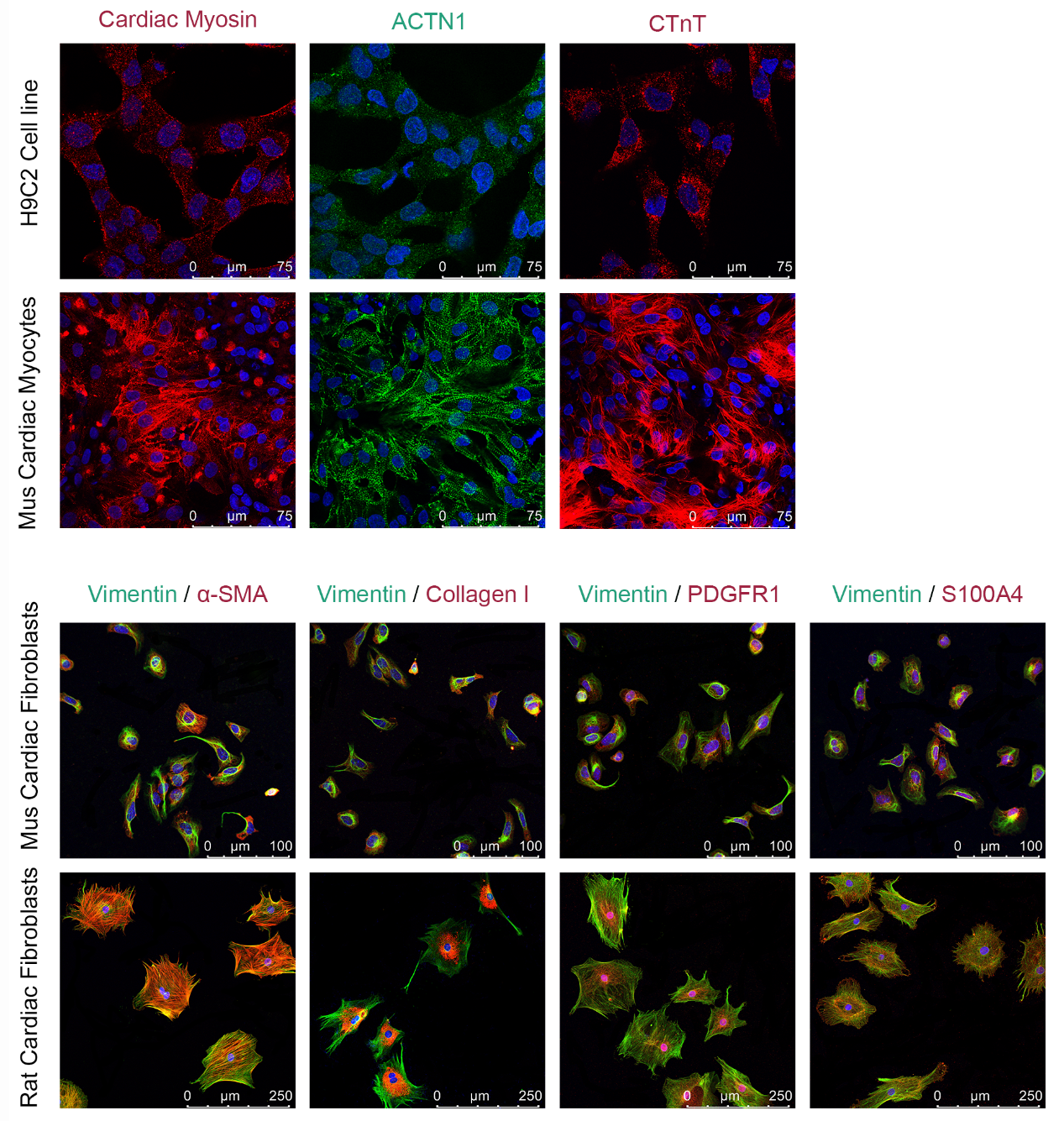


**Supplemental Figure 3.** Representative immunofluorescence images of primary mouse CMs and H9C2 cell lines for the staining of anti-cardiac myosin, anti-Alpha Actinin (ATCN1) and anti-cardiac troponin t (CTnT), as well as immunofluorescence images of primary mouse CFs and rat CFs for the staining of anti-Vimentin, anti-Collagen I, anti-α-SMA, anti-PDGFR1 and anti-S100A4. The fact that the majority of isolated CMs were cardiac myosin^+^, ACTN1 ^+^ and CTnT^+^, while the majority of isolated CFs were Vimentin ^+^, α-SMA ^+^, Collagen I ^+^, PDGFR1^+^ and S100A4^+^ verified the purity of isolated primary cells used for our *in vitro* study.

**Supplemental Figure 4**

**
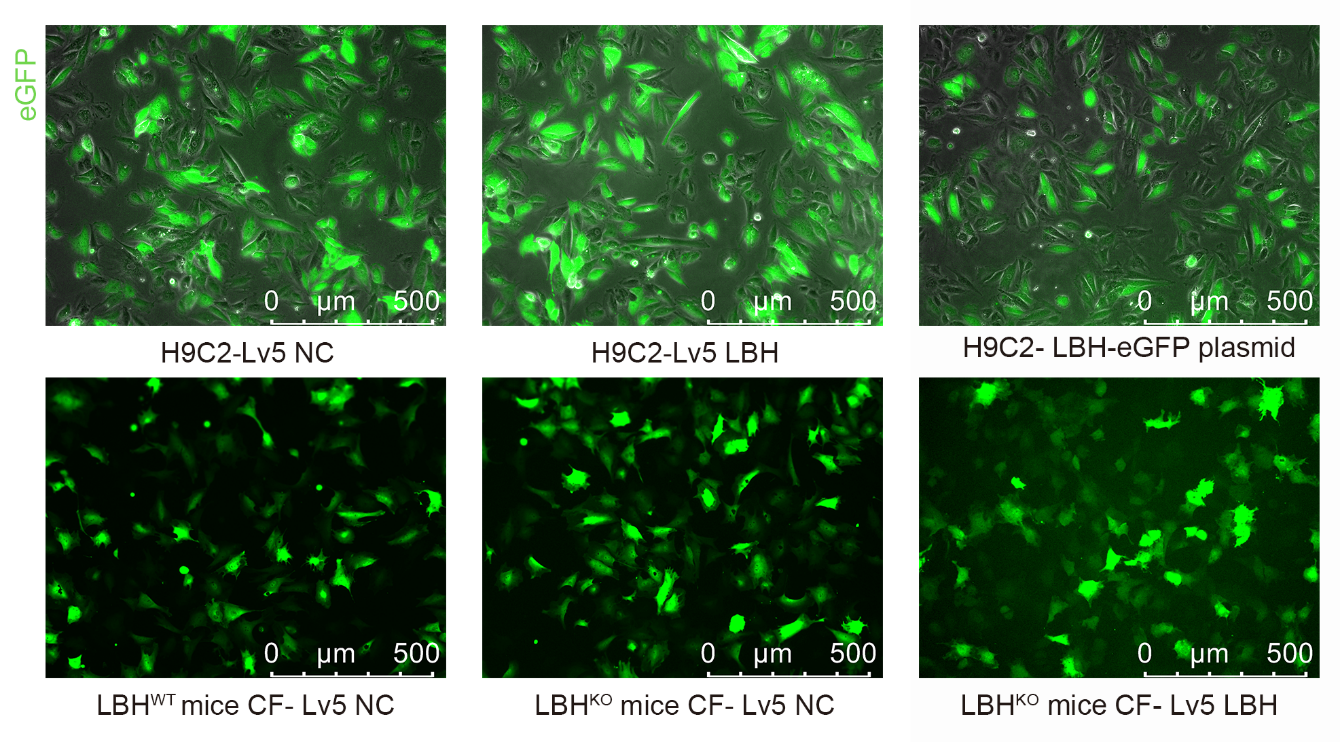
**

**Supplemental Figure 4.** Immunofluorescence images indicating the infection efficiencies of puromycin-screened H9C2 cell lines prepared for the following experiments. For mouse CF infection by lentivirus, optimized multiplicity of infection (MOI), 50:1, were gained from gradient tests. Representative images of GFP fluorescence indicated sufficient infection efficiencies were achieved under this MOI, for all types of lentiviruses we applied in this research.

**Supplemental Figure 5**

**
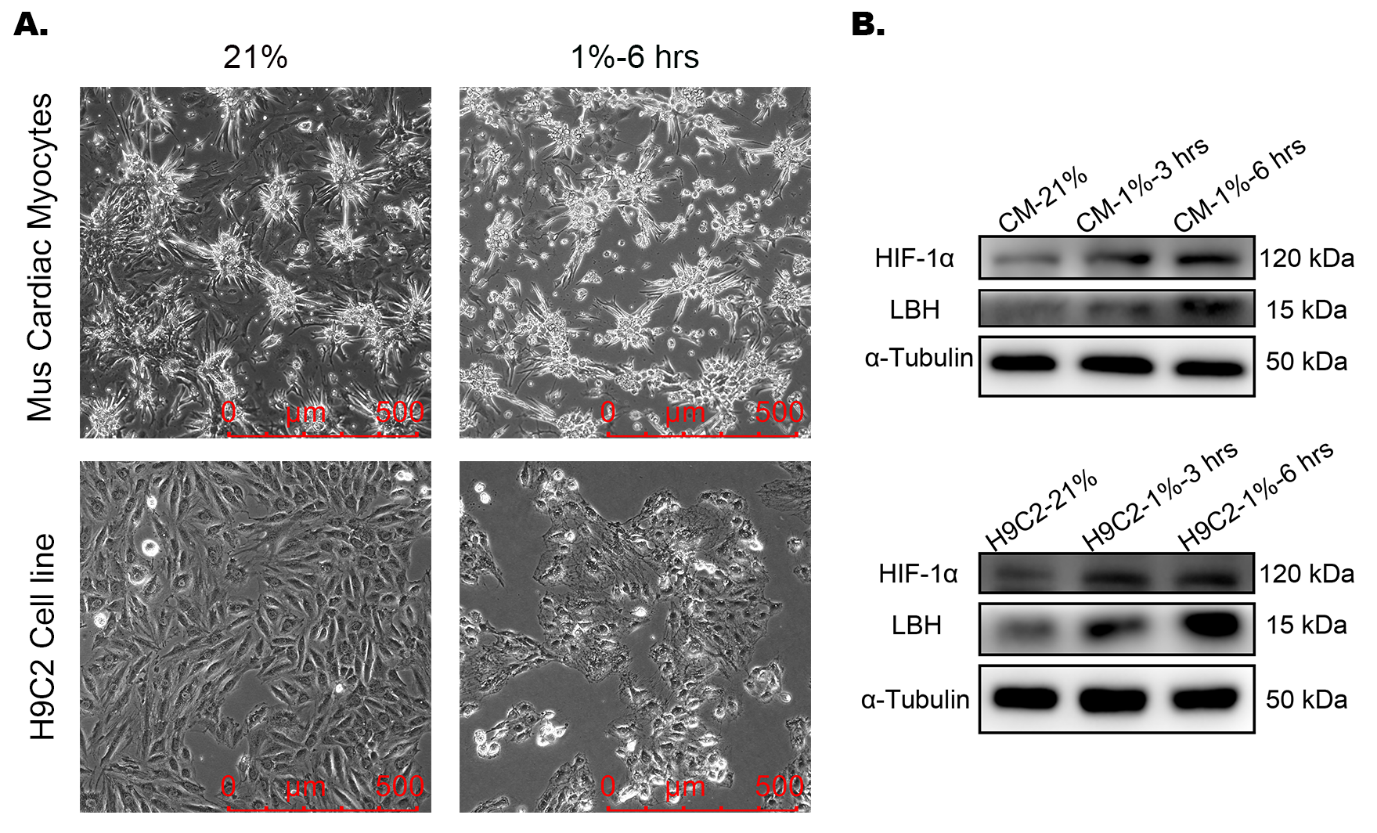
**

**Supplemental Figure 5.** **(A)** Representative images of H9C2 cells and mouse CMs for phase contrast imaging. The results showed that 6 hours of hypoxia caused significant cellular shrinkage and deformation in both H9C2 cells and CMs. **(B)** Representative image of Western blotting detecting protein levels of LBH and HIF-1α in H9C2 cells and mouse CMs under hypoxia (1% O_2_).

**Supplemental Figure 6**

**
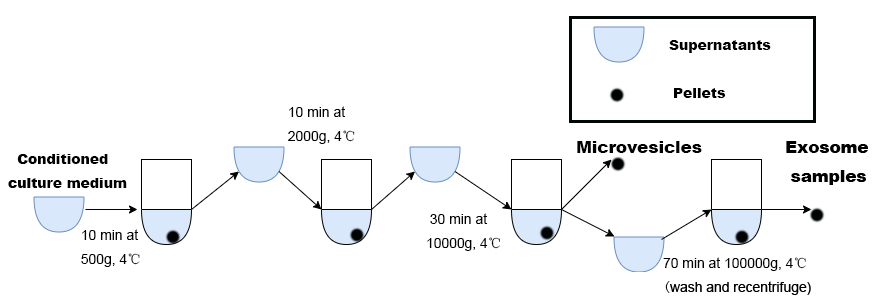
**

**Supplemental Figure 6.** Protocols of exosome isolation by differential ultracentrifugation used in this study, presented as flowchart.

**Supplemental Figure 7**

**
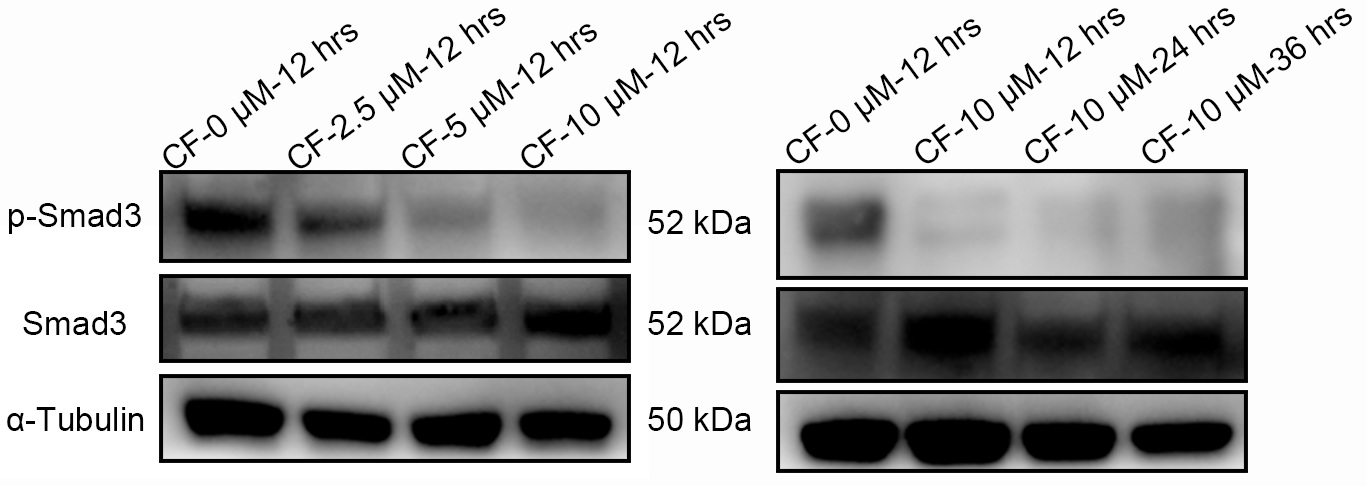
**

**Supplemental Figure 7.** **(A)** The time length gradient test for mouse CFs treated with inhibitor SIS3. **(B)** The concentration gradient test for mouse CFs treated with inhibitor SIS3. These data showed that SIS3 at 10 μM is sufficient enough to achieve and sustain significant suppression of Smad3 phosphorylation in mouse CFs during 36 hours.

**Supplemental Figure 8**


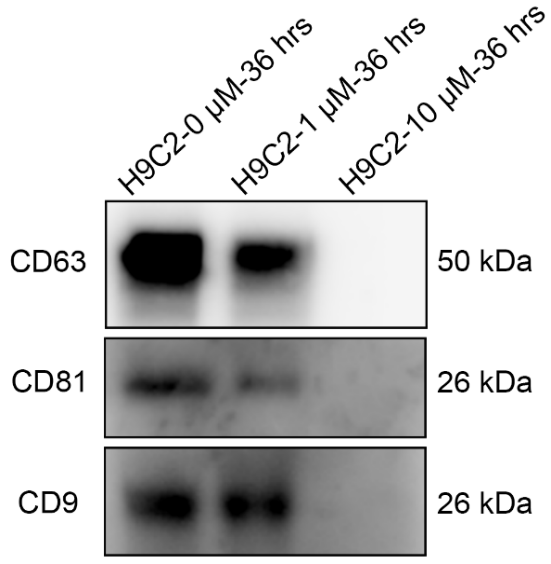


**Supplemental Figure 8.** The concentration gradient test for H9C2 cells treated with inhibitor GW4869. The WB result of exosome samples isolated from culture medium of H9C2 cells treated with different inhibitor concentrations showed that GW4869 at 10 μM is sufficient enough to achieve and sustain significant inhibition of exosome production and secretion in H9C2 cells during 36 hours, which is equivalent to the entire co-culture process.

**Supplemental Figure 9**

**
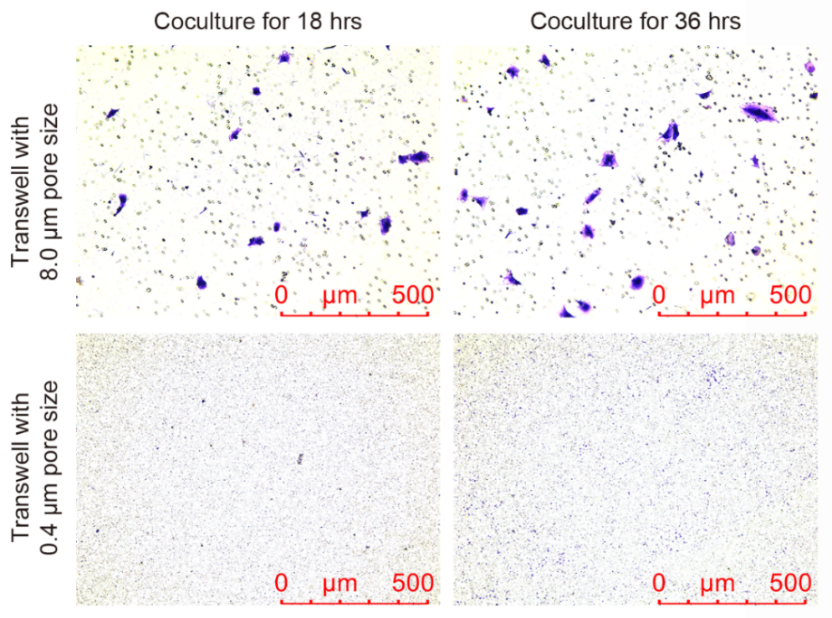
**

**Supplemental Figure 9.** The crystal violet staining of transwell inserts used for coculture. It showed that although some H9C2 cells migrated through the membranes of insert with 8.0 μm pore size, they were not unable to migrated through the membranes of insert with 0.4 μm pore size during the entire process, which were actually applied for the coculture of LBH-eGFP transfected H9C2 cells and rat CFs, thus ensured that LBH-eGFP detected in CFs was transferred through intercellular communication without cell-to-cell contact.

**Supplemental Figure 10**

**
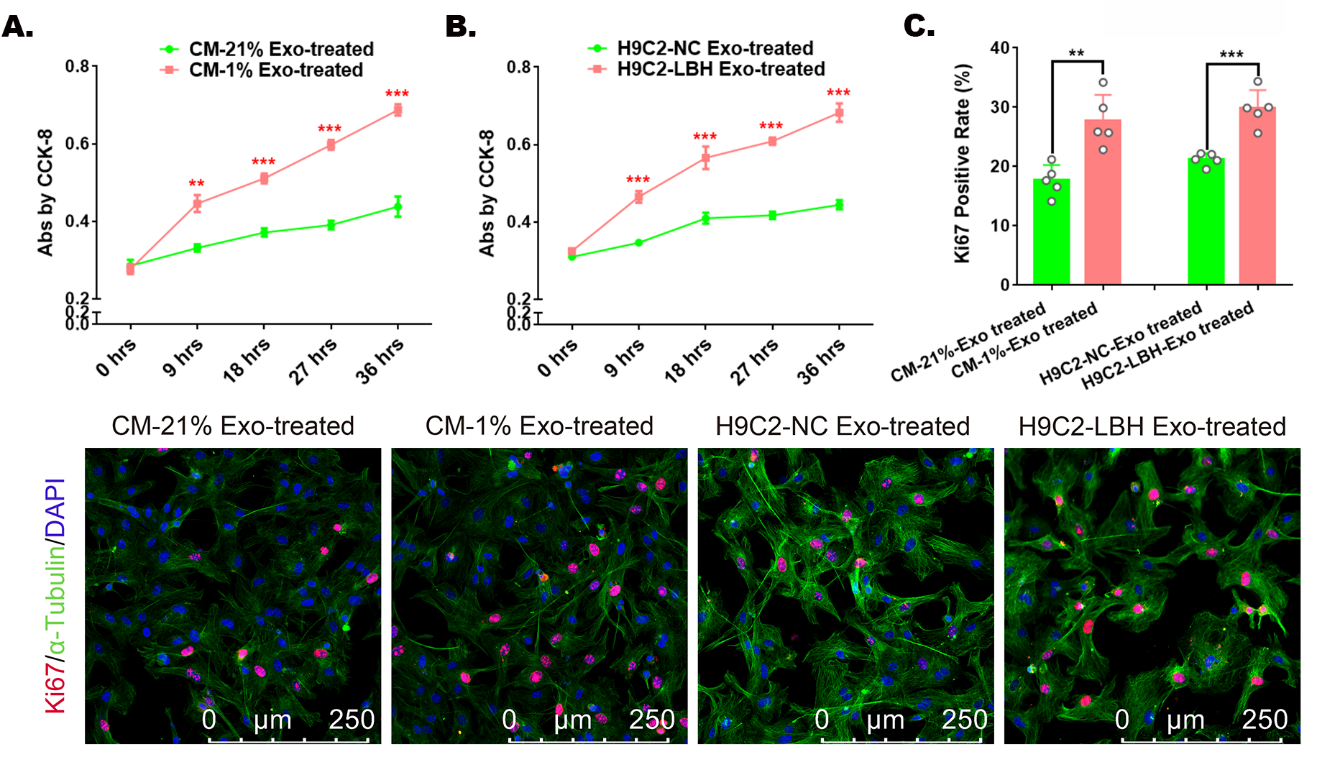
**

**Supplemental Figure 10.** The effects of CM-derived exosomes on CFs proliferation indicated by EdU staining were verified by CCK-8 assay and anti-Ki67 staining. **(A)** OD_450 nm_ of CM-hypoxia exosomes treated mouse CFs detected by CCK-8 assay, and corresponding statistical analysis (***p<0.001 vs. CM-21%-Exo treated). **(B)** OD_450_ nm of H9C2-LBH exosomes treated rat CFs detected by CCK-8 assay, and corresponding statistical analysis (***p<0.001 vs. H9C2-NC-Exo treated). **(C)** Representative immunofluorescence images of anti-Ki67 staining of mouse CFs treated with CM-hypoxia exosomes or rat CFs treated with H9C2-LBH exosomes, and corresponding statistical analysis of Ki67 positive rates (**p<0.01, ***p<0.001 vs. CM-21%-Exo treated / H9C2-NC-Exo treated).

**Supplemental Figure 11**


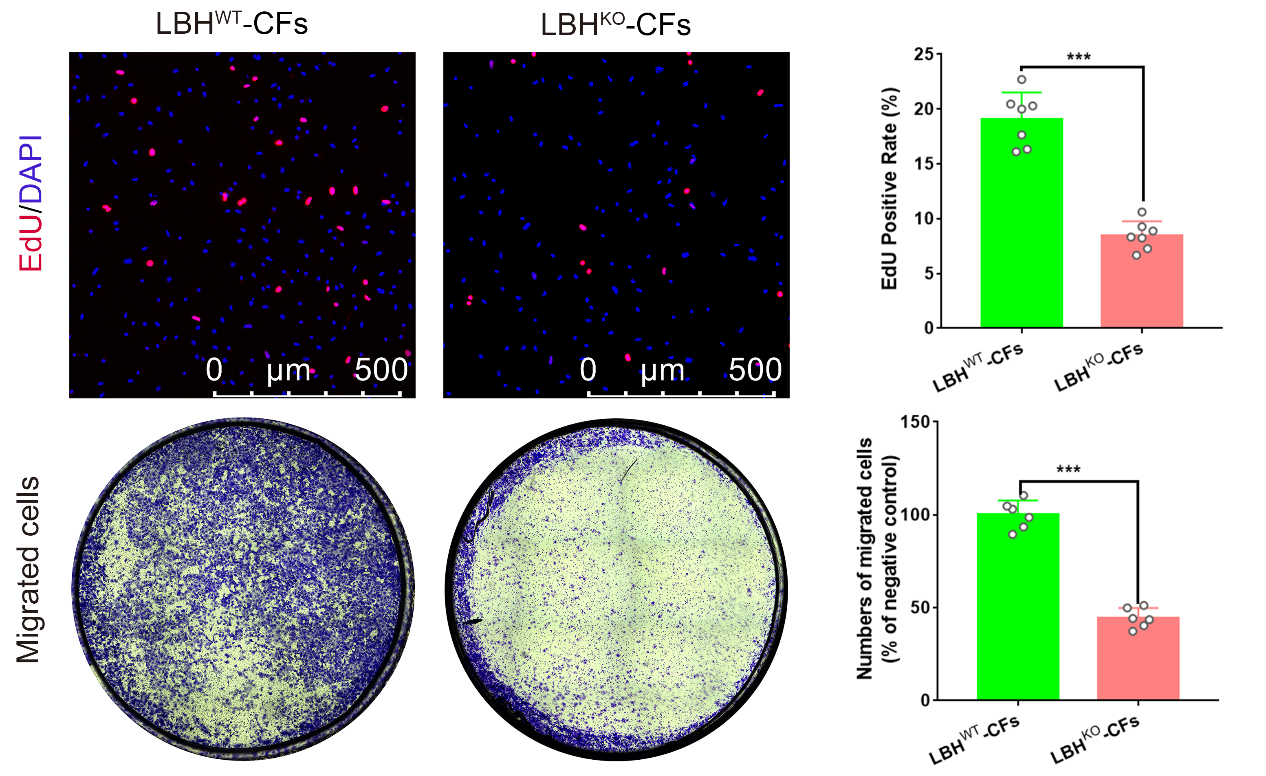


**Supplemental Figure 11.** The effects of LBH gene on CFs proliferation and migration were verified by LBH knockout. **(A)** EdU staining of CFs isolated from LBH^WT^ and LBH^KO^ mice, and corresponding statistical analysis (***p<0.001 vs. LBH^WT^-CFs). **(B)** Representative images of the Transwell assay of CFs isolated from LBH^WT^ and LBH^KO^ mice, and corresponding statistical analysis (***p<0.001 vs. LBH^WT^-CFs).

**Supplemental Figure 12**

**
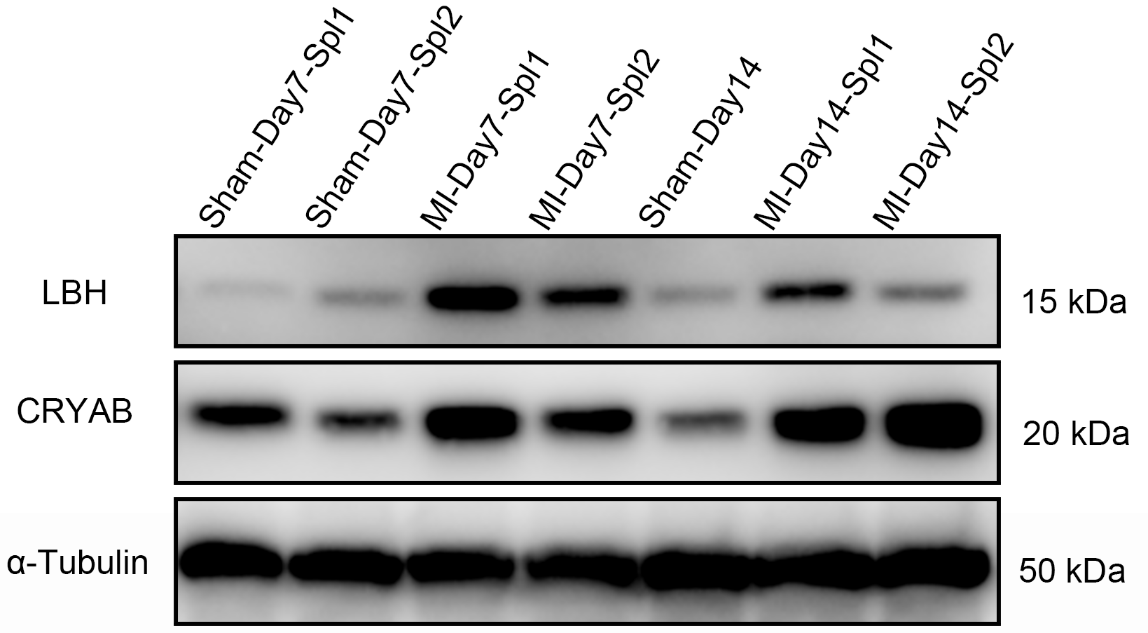
**

**Supplemental Figure 12.** Protein expression levels of LBH and CRYAB of peri-infarction heart tissue at the indicated time points during chronic cardiac fibrosis induced by the coronary artery ligation. The fact that upregulated LBH started to decline during the maturation phase while upregulated CRYAB remained stable agreed with the same results presented in our previous study.

**Supplemental Figure 13**


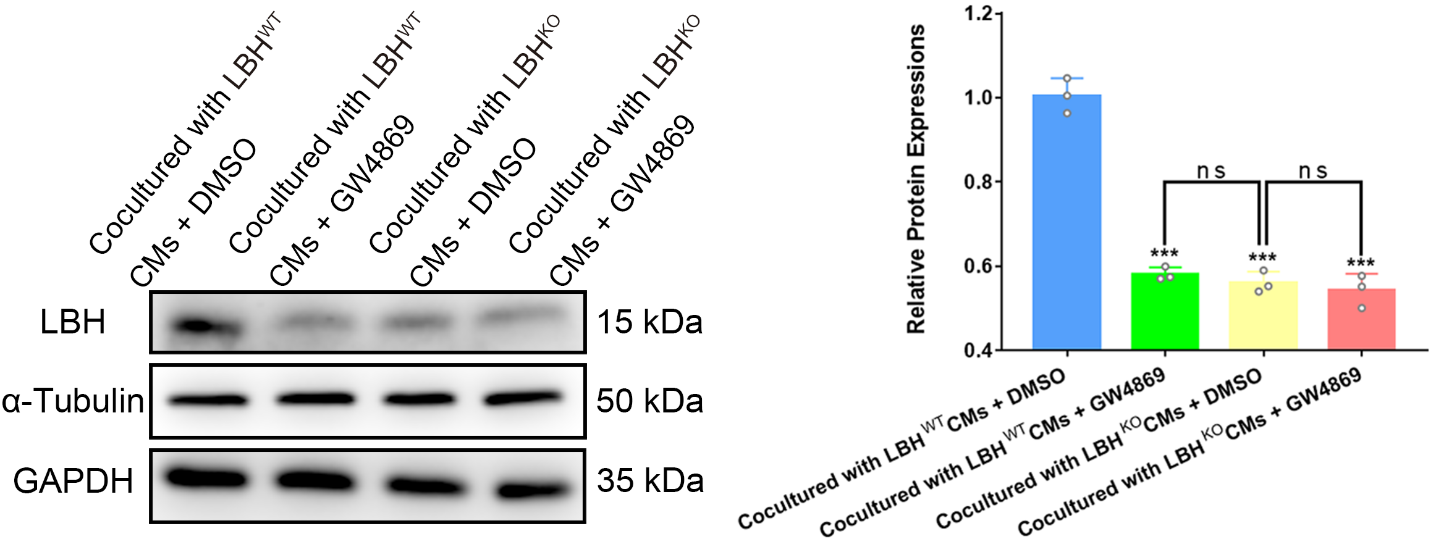


**Supplemental Figure 13.** Protein expression levels of LBH in LBH^WT^ CFs cocultured with LBH^WT^/ LBH^KO^ CMs under the treatment with inhibitor GW4869 (***p<0.001 vs. CFs cocultured with LBH^WT^CMs+DMSO). The fact that upregulated LBH was observed in CFs cocultured with LBH^WT^ CMs without he treatment with inhibitor GW4869 confirmed our assumption that LBH protein in CMs was transferred into CFs by exosome secretion and subsequently activated CF activation by increasing the LBH levels in CFs as exosome recipient cells.

**Supplemental Figure 14**


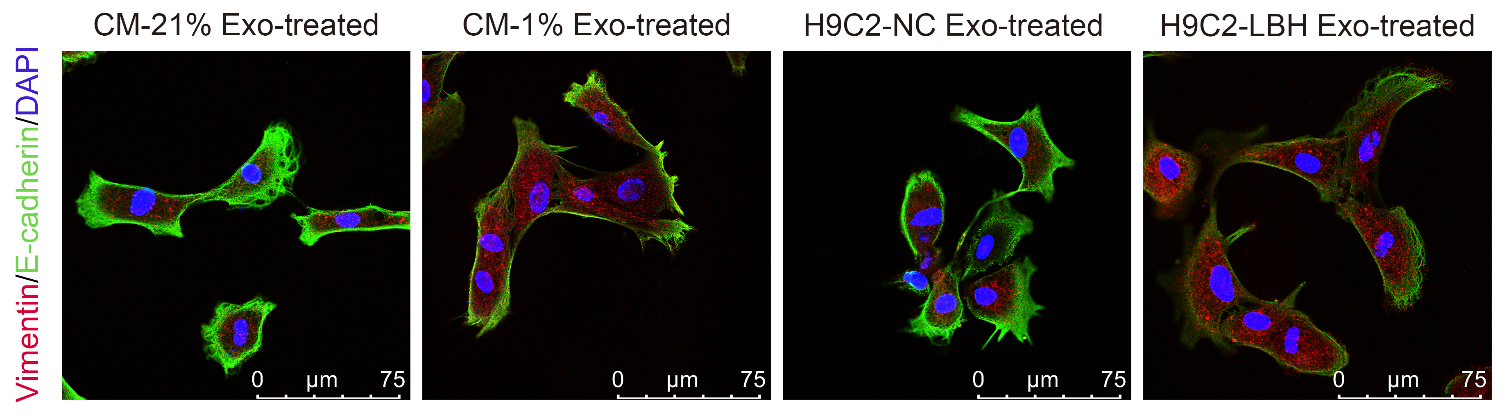


**Supplemental Figure 14.** Representative immunofluorescence images of vimentin and E-cadherin expression in mouse CFs treated with hypoxic CM-derived exosomes and in rat CFs treated with H9C2-LBH-derived exosomes. The E-cadherin downregulation, together with vimentin upregulation in LBH+ exosome-treated CFs, were in accordance with WB results, uniformly indicated the EMT-like process in CFs after LBH+ exosomes treatment.
